# Expected contraction in the distribution ranges of demersal fish of high economic value in the Mediterranean and European Seas

**DOI:** 10.1038/s41598-022-14151-8

**Published:** 2022-06-16

**Authors:** Emna Ben Lamine, Alexandre Schickele, Eric Goberville, Gregory Beaugrand, Denis Allemand, Virginie Raybaud

**Affiliations:** 1 Université Côte d’Azur, CNRS, UMR 7035 ECOSEAS, Nice, France; 2 LIA ROPSE, Laboratoire International Associé Université Côte d’Azur—Centre Scientifique de Monaco, Monaco, Monaco; 3 Sorbonne Université, CNRS, UMR 7093 LOV, Villefranche-sur-Mer, France; 4 Muséum National d’Histoire Naturelle, Sorbonne Université, Université de Caen Normandie, Université Des Antilles, CNRS, UMR 8067 BOREA, Paris, France; 5 Université Lille, Université Littoral Côte d’Opale, CNRS, UMR 8187 LOG, Wimereux, France; 6 Centre Scientifique de Monaco, Monaco, Monaco

**Keywords:** Ecology, Environmental sciences

## Abstract

Fisheries and aquaculture are facing many challenges worldwide, especially adaptation to climate change. Investigating future distributional changes of largely harvested species has become an extensive research topic, aiming at providing realistic ecological scenarios on which to build management measures, to help fisheries and aquaculture adapt to future climate-driven changes. Here, we use an ensemble modelling approach to estimate the contemporary and future distributional range of eight demersal fish species of high economic value in the Mediterranean Sea. We identify a cardinal influence of (i) temperature on fish species distributions, all being shaped by yearly mean and seasonality in sea bottom temperature, and (ii) the primary production. By assessing the effects of changes in future climate conditions under three Representative Concentration Pathway (RCP2.6, RCP4.5 and RCP8.5) scenarios over three periods of the twenty-first century, we project a contraction of the distributional range of the eight species in the Mediterranean Sea, with a general biogeographical displacement towards the North European coasts. This will help anticipating changes in future catch potential in a warmer world, which is expected to have substantial economic consequences for Mediterranean fisheries.

## Introduction

Our oceans are getting warmer, less oxygenated and more acid^1^, altering the growth, survival, and productivity rates of marine fish organisms, leading to distributional shifts^2^, ensuing changes in potential fish catch availability to fisheries^3^. This sensitivity of species to changing environmental conditions is triggering complex ecological, conservation and management challenges^4^, considering not only the direct response of individual and populations to physical, chemical and climate conditions, but also indirect responses through alterations in species interactions, community changes, and their consequences at the ecosystem and socio-economic levels^5^. Projecting the expected response of commercial fish species to climate change is therefore crucial for ensuring food security and sustainable resource management^6^, especially for countries relying on fisheries protein supply^7^.

The Mediterranean Sea, the largest semi-enclosed sea in the world, is a biodiversity hotspot with a high endemism rate (about 20%^8^), and a receptacle for exotic species^9^. Depending on the phylum considered, marine organisms in the Mediterranean Sea represent 4–18% of the world marine biodiversity^10^. However, the Mediterranean Sea is under considerable threat from the combined effects of anthropogenic pressures (*e.g.,* pollution, overfishing) and rapid warming, sea surface temperatures increasing two to three times faster than the global ocean^11^. In contrast and in line with global trends, fish farming has become as productive as wild fishing in the Mediterranean Sea over the recent decades, both quantitatively (in landing) and commercially (in revenue)^12^.

Although based on simplifying assumptions—such as species niche conservatism^13^ or the equilibrium hypothesis^14^—Species Distribution Models (SDMs) are popular statistical tools built by correlatively linking observed species distributions and environmental data^15^ to assess the past, present or future spatial distribution of a species of interest, for conservation, fisheries, or aquaculture management purposes^16,17^. When combined with a multi-GCMs (General Circulation Models) and multi-IPCC (Intergovernmental Panel on Climate Change) emissions scenarios approach, ensemble modelling computed from a large range of modelling algorithms (multi-SDMs) is the best-practice needed in biodiversity assessments to capture (i) the variability related to the ecological niche estimation and (ii) uncertainties from future climate projections^18^.

Here, we focus on demersal fish species of high economic value i.e., the eight commercial fish species with the highest total economic value of landings in the Mediterranean and the Black Sea, according to the Food and Agriculture Organization^12^. While most of the previous studies on these species investigated distributional changes at a local scale—or at the scale of a scientific survey^8^—we aimed to address current knowledge gaps in both their present and future spatial distributions over the whole Mediterranean and European Seas. Considering that^19^ recently investigated changes in the distribution of Mediterranean small pelagic fish, we focused here on fish species representing more than 32% of total Mediterranean landing value^12^: the surmullet *Mullus surmuletus*, the red mullet *Mullus barbatus*, the European hake *Merluccius merluccius*, the common sole *Solea solea*, the common pandora *Pagellus erythrinus*, and the anglerfish *Lophius* spp. (*Lophius budegassa* and *Lophius piscatorius*). We processed the two anglerfish species as one group: both species are extremely similar morphologically^20^, leading to difficulties in splitting in fisheries statistics^8^. We also included the gilthead seabream *Sparus aurata* and the European seabass *Dicentrarchus labrax* that represent 33% and 27% of the total Mediterranean aquaculture, respectively^12,21^.

For the eight species, we examined long-term and large-scale distributional range projections under three RCP scenarios—RCP2.6, RCP4.5 and RCP8.5—using an ensemble modelling approach^9,22^. We then evaluated predicted changes in species’ environmental suitability at a manageable level, i.e., for each Mediterranean Exclusive Economic Zone. By estimating changes in future environmental suitability per EEZ by the end of the century, we stress that SDMs provide a relevant and reliable basis for ensuring effective fisheries management and for supporting conservation plan in the most exposed Mediterranean regions^23^.

## Results

### Species distributions models and environmental variables

**Figure 1:**
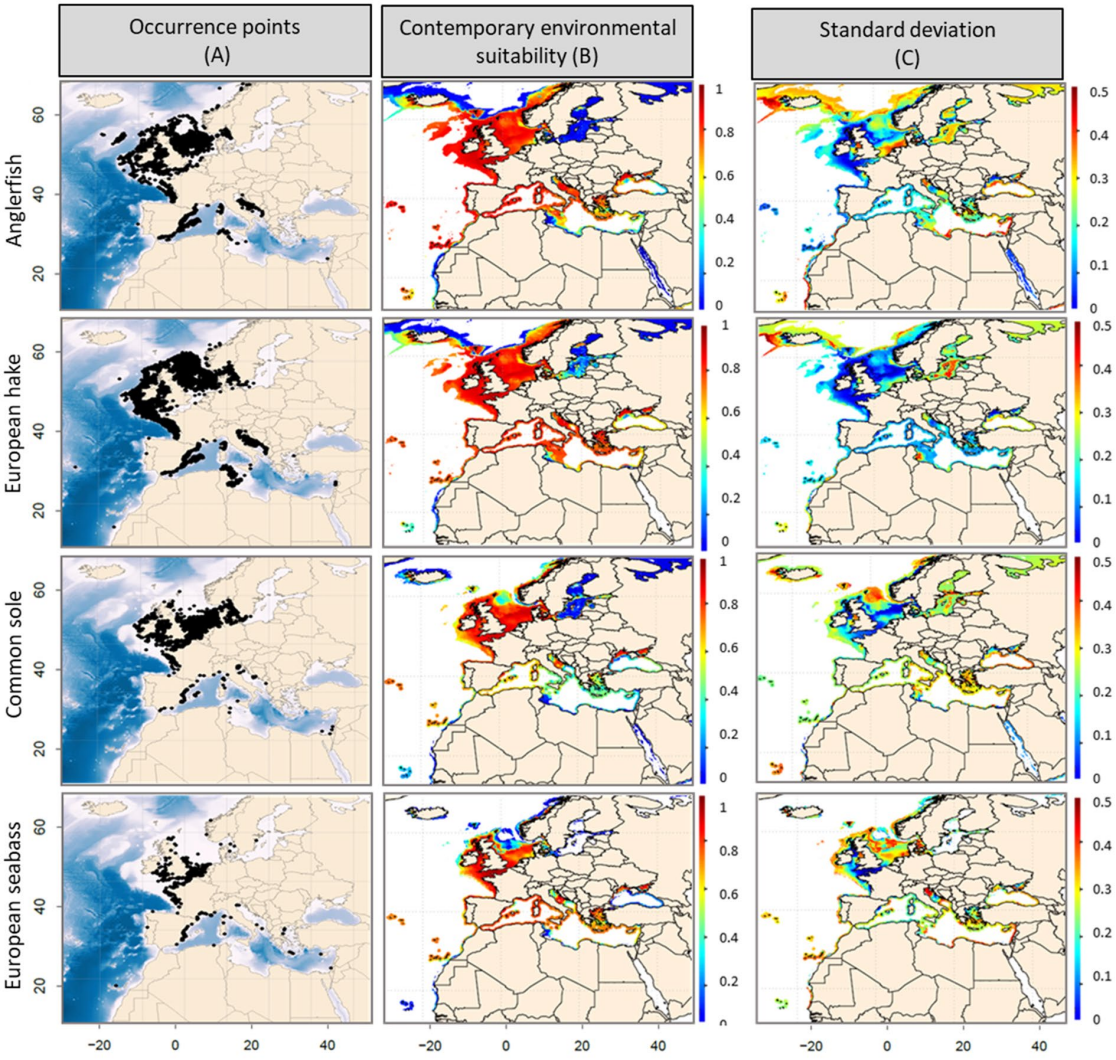
(**A**) Contemporary (1990–2017) observed distribution, (**B**) modelled environmental suitability index (from 0 to 1) and (**C**) associated standard deviation, based on the set of retained algorithms and cross-validation runs performed for anglerfish, European hake, common sole and the European seabass.

Based on both Continuous Boyce Index (CBI) values and the examination of species response curves (i.e. ecological significancy and low inter-algorithm divergence; supplementary material 1 & 2), we retained the algorithms that reproduce the best species distributions (Table 1): for each ensemble model and fish species, the minimum number of models retained was 3 (for the European hake) and the higher was 4 (*e.g.,* for the remaining species). Whatever the species, the MARS and NPPEN algorithms were always retained (Supplementary material 1). Sea Surface Temperature (SST), seasonal (SSTr) and monthly (SSTvar) variations, and salinity (SSS), did not contribute substantially to the construction of our models. The three most contributing parameters, independently of the algorithm, were (i) mean Sea Bottom Temperature (SBT), (ii) mean annual Sea Bottom Temperature range (SBTr; seasonal variability), and (iii) primary production (Log_PP). Despite their high pairwise correlation (r = 0.80; Supplementary Material 3), seasonal variability in Sea Bottom Temperature (SBTr) and mean monthly Sea Bottom Temperature variance (SBTvar)—a proxy of short-term climatic variability—have dissimilar ecological influences. Models built using SBTr were more likely to reproduce observed species geographical distributions. We then used the models retained by the numerical procedure—in combination with the most contributing variables—to reproduce the contemporary geographical distributions of each species (Fig. 1).

**Table 1:** Environmental variables and SDM retained after application of our modelling procedure.

Species	Variables and algorithm selected	
Anglerfishes *Lophius* spp.	Variables	SBT, SBTr, Log_PP
	Algorithms*	ANN, FDA, MARS, NPPEN
	CBI (mean)	0.816
Surmullet *Mullus surmuletus*	Variables	SBT, SBTr, Log_PP
	Algorithms	ANN, GAM, MARS, NPPEN
	CBI (mean)	0.868
Red mullet *Mullus barbatus*	Variables	SBT, SBTr, Log_PP
	Algorithms	GAM, FDA, MARS, NPPEN
	CBI (mean)	0.856
European hake *Merluccius merluccius*	Variables	SBT, SBTr, Log_PP
	Algorithms	GAM, MARS, NPPEN
	CBI (mean)	0.879
Common sole *Solea solea*	Variables	SBT, SBTr, Log_PP
	Algorithms	ANN, FDA, MARS, NPPEN
	CBI (mean)	0.835
Common pandora *Pagellus erythrinus*	Variables	SBT, SBTr, Log_PP
	Algorithms	GAM, FDA, MARS, NPPEN
	CBI (mean)	0.827
European seabass *Dicentrarchus labrax*	Variables	SBT, SBTr, Log_PP
	Algorithms	ANN, MARS, NPPEN
	CBI (mean)	0.822
Gilthead seabream *Sparus aurata*	Variables	SBT, SBTr, Log_PP
	Algorithms	ANN, FDA, MARS, NPPEN
	CBI (mean)	0.823

*SBT* Sea Bottom Temperature, *SBTr* annual range of Sea Bottom Temperature, *log_PP* log-transformed Primary Production, *GAM* Generalised Additive Model, *ANN* Artificial Neural Network, *FDA* Flexible Discriminant Analysis, *MARS* Multiple Adaptive Regression Splines, *NPPEN* Non-Parametric Probabilistic Ecological Niche model.

*The selected SDMs had a Continuous Boyce Index CBI > 0.5 and satisfying response curves.

### Contemporary environmental suitability

**Figure 2:**
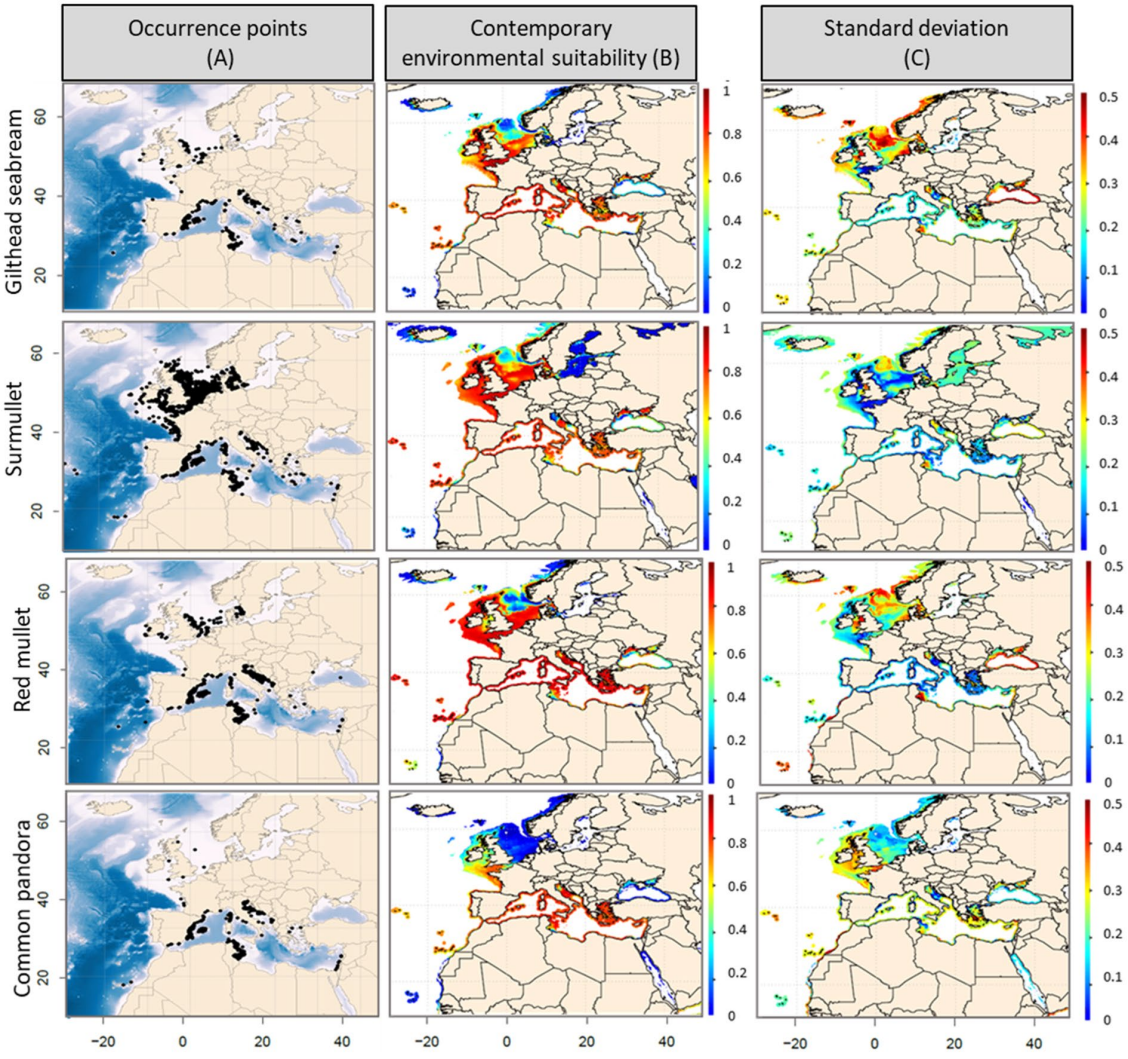
(**A**) Contemporary (1990–2017) observed distribution, (**B**) modelled environmental suitability index (from 0 to 1) and (**C**) associated standard deviation, based on the set of retained algorithms and cross-validation runs performed for gilthead seabream, surmullet, red mullet and common pandora.

The contemporary spatial range (1990–2017) of all species were reproduced well by our models (Fig. 1, 2A versus Fig. 1, 2B), except in the Black Sea and along the Mauritanian, Moroccan and Algerian coasts, where predicted Environmental Suitability Index (ESI) values varied between 0.4 and 0.8, while no occurrence was reported. Such discrepancies may result from species under-sampling in Northern African countries, from local factors—such as the way in which biotic interactions can shape realized assemblages of species despite suitable environmental conditions—and/or from possible limiting environmental drivers, such as oxygen, nutrients or pH, not included in our simulations because of data availability at the time of the analysis and/or at a macroecological scale.

The highest ESI values (> 0.8; Figs. 1B and 2B) over the period 1990–2017 were modelled in the Mediterranean, Celtic and North Seas, but for common sole (ESI between 0.2 and 0.6 in the Mediterranean Sea) and common pandora (ESI < 0.2 in the North Sea). According to our models, only two species had suitable habitat up to Scandinavian coasts: anglerfishes and European hake. For all species, ESIs ranged from 0.4 to 0.8 in the Black Sea and the Moroccan Atlantic coasts and were lower in the Baltic Sea and the Mauritanian Sea (ESI < 0.4).

For all species, our projections showed medium to low SD in the Mediterranean Sea, with values ranging from 0.1 to 0.5 (Figs. 1, 2C). This suggests an overall spatial convergence of our simulations based on a multi-SDM framework. Our models showed higher (~ 0.5) standard deviation (SD) values in geographical cells that correspond to intermediate or low ESI values, suggesting a lower convergence among algorithms at the edge of spatial range (e.g., Black Sea, Moroccan Atlantic Coasts, and the Baltic Sea) due to the variability in environmental conditions.

### Future environmental suitability

**Figure 3:**
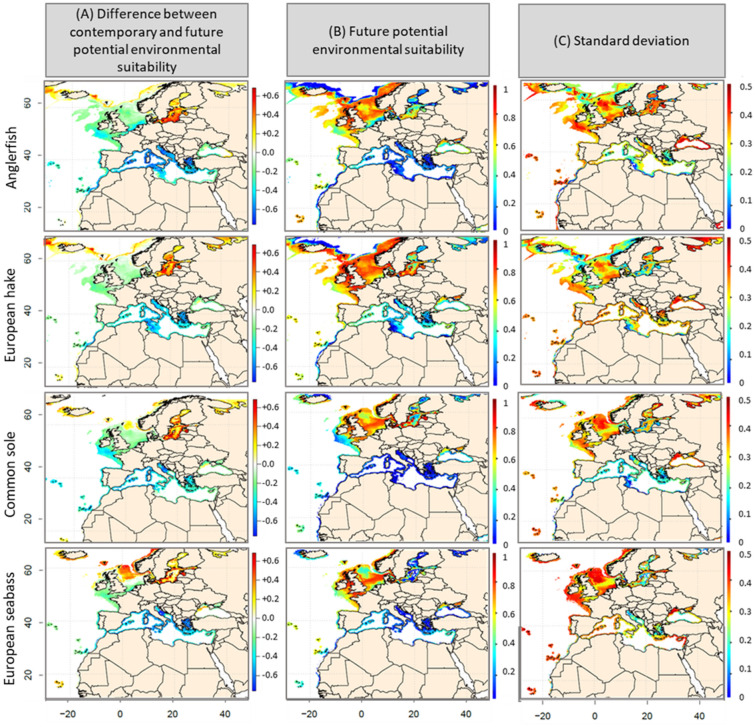
(**A**) Differences in Environmental Suitability Index (ESI) values calculated between the current period (1990–2017) and the decade 2090–2099, under scenario RCP8.5. (**B**) Modelled ESI for anglerfish, European hake, common sole, European seabass, over the period 2090–2099, under scenario RCP8.5. (**C**) Standard deviation based on 50 simulations per algorithm selected in the ensemble model (i.e., 10 cross-validation runs × 5 general circulation models per algorithm).

**Figure 4:**
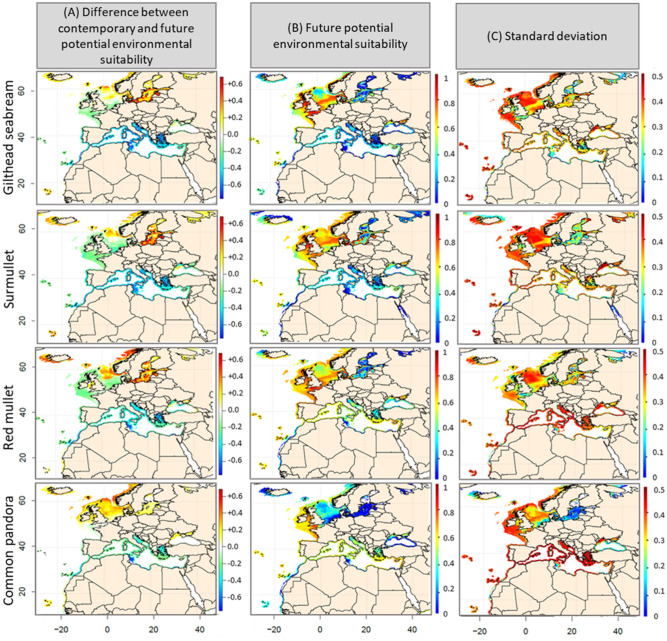
(**A**) Differences in Environmental Suitability Index (ESI) values calculated between the current period (1990–2017) and the decade 2090–2099, under scenario RCP8.5. (**B**) Modelled ESI for gilthead seabream, surmullet, red mullet, common pandora over the period 2090–2099, under scenario RCP8.5. (**C**) Standard deviation based on 50 simulations per algorithm selected in the ensemble model (i.e., 10 cross-validation runs × 5 general circulation models per algorithm).

For each of the eight studied species, distributional ranges under the scenario Representative Concentration Pathway (RCP) 8.5 conditions for the end of the century (2090–2099), and both their standard deviations and differences between contemporary and future distributions, are detailed in Figs. 3, 4 (B, C and A, respectively). Other scenarios and periods are provided in Supplementary Material 4.

For all species, a decrease in ESI values between the contemporary period (1990–2017) and the last decade of the century (2090–2099) was projected in the Mediterranean and the Black Seas, as well as along the Mauritanian coasts (Fig. 3A and 4A and Supplementary Material 4). This predicted decrease ranged from − 0.2 to − 0.4 (RCP2.6) and from − 0.2 to − 0.6 (RCP4.5 and 8.5). An increase in ESI values (+ 0.2 under RCP4.5 and + 0.6 under RCP8.5) was projected in the North Sea for European seabass, red mullet, surmullet and common pandora. For the other species, ESIs are likely to decrease between − 0.2 (RCP4.5) and − 0.4 (RCP8.5) in the North Sea. In the Baltic Sea, all species ESI are likely to increase by 0.2 (RCP4.5) and by 0.2 to 0.6 (RCP8.5). The biogeographical peculiarities of the Baltic Sea—with a strong mixture of marine, brackish, and freshwater conditions^24^—impel us to interpret these projections with caution. For the end of the century and RCP8.5 (Fig. 2B), very low (< 0.4) ESI values were projected in the Mediterranean Sea for all species, except for red mullet and common pandora for which ESIs range from 0.4 to 0.6 by 2090–2099. Predicted ESIs were high (0.6 to 1) in the Celtic and North Seas for all species, suggesting a northward species distributional range shift. While projected ESIs for common pandora ranged between 0.5 and 0.7 in the Celtic Sea, values did not exceed 0.4 in the North Sea and at higher latitudes, suggesting an absence of highly suitable conditions in these regions. Our results show a clear convergence among projections in the Mediterranean, Celtic and North Seas, with a low to medium SD (between 0.3 and 0.5) for all species, but red mullet and common pandora (SD > 0.5; Figs. 3, 4C). For all future time periods, the loss in species spatial coverage clearly depend on the level of warming (Table 2). The projected variation of the spatial coverage in comparison to the contemporary period that we calculated for each species showed a decline ranging from − 16.09% to − 53.01%. European hake is the least impacted species in terms of predicted spatial extent (− 21.76% under RCP8.5) as opposed to common pandora (− 53%). Anglerfishes, gilthead seabream and common pandora will lose more than 30% of their potential spatial coverage by the end of the century under all scenarios (Table 2).

**Table 2:**
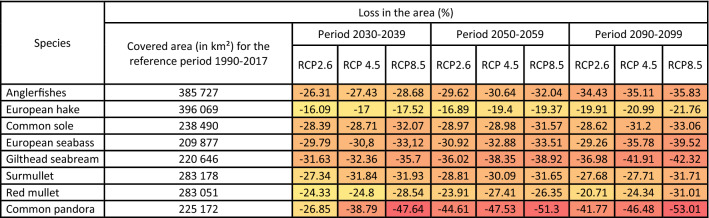
Projected loss in the geographical distribution areas of the eight fish species, expressed in percentage in comparison with the reference period 1990–2017, under RCP scenarios 2.6, 4.5 and 8.5, and for three future periods: 2030–2039, 2050–2059 and 2090–2099. Low (high) values are in yellow (red).

### Climatic range shifts in exclusive economic zones (EEZs)

**Figure 5:**
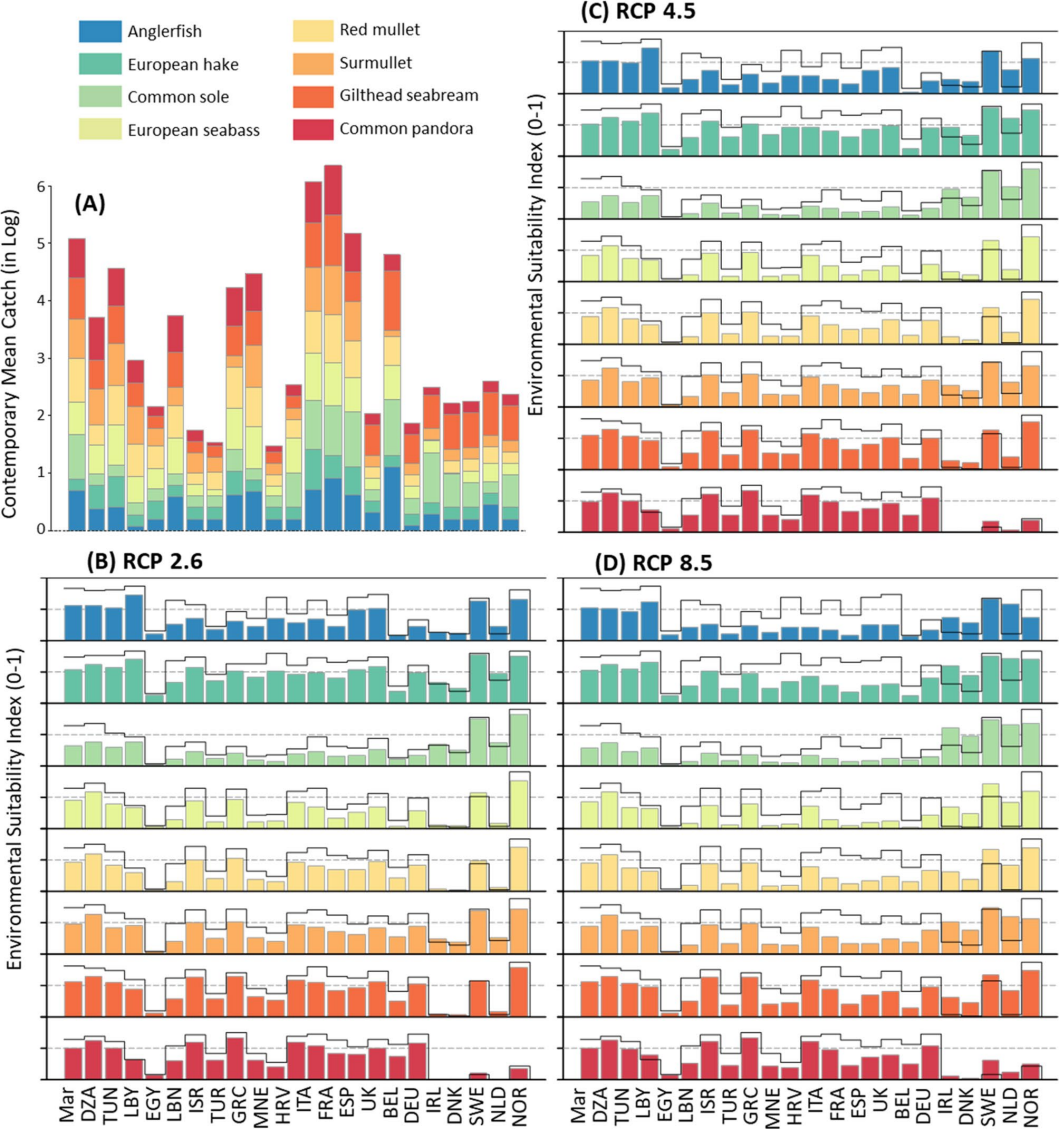
(**A**) Contemporary (1990–2017) mean catch (in log) for each studied species in the Mediterranean Sea. (**B**–**D**) Projected changes in the Environmental Suitability Index (ESI) per Exclusive Economic Zone (EEZ) for all species, for the end of the century (2090–2099) under RCP2.6 (**B**; bottom left), RCP4.5 (**C**; top right) and RCP8.5 (**D**; bottom right) scenarios. Bar plots for ESI are scaled from 0 to 1, the full black line corresponds to the ESI values for the current period (1990–2017) and colored bar correspond to the ESI values projected for 2090–2099. Countries with catches under 1000 tons per year are not shown. Countries are: *MAR* Morocco, *DZA* Algeria, *TUN* Tunisia, *LBY* Libya, *EGY* Egypt, *LBN* Lebanon, *ISR* Israel, *TUR* Turkey, *GRC* Greece, *MNE* Montenegro, *HRV* Croatia, *ITA* Italy, *FRA* France, *ESP* Spain, *UK* United Kingdom, *BEL* Belgium, *DEU* Germany, *IRL* Ireland, *DNK* Denmark, *SWE* Sweden, *NLD* Netherlands, *NOR* Norway.

Anglerfish, European hake, common sole and European sea bass—species of major importance in the European Atlantic and the Mediterranean fisheries, especially along the coasts of the United Kingdom and Norway—were mostly captured along the European coasts (Fig. 5A), i.e., in EEZs characterized by high contemporary (1990–2017) and future ESI values, even for a pronounced warming (Fig. 5; supplementary material 5). For these four species and by the end of the century, we projected a decrease in ESI values in the Mediterranean EEZs (from − 0.2 to − 0.4 under scenarios RCP2.6 and RCP8.5, respectively; Fig. 5; supplementary material 5).

Gilthead seabream, surmullet, red mullet and common pandora are mainly harvested in the Mediterranean countries where high ESI values were observed over the period 1990–2017. While the ESI values for these four species are expected to decrease in the Mediterranean EEZs by the end of the century for all future periods (Fig. 5; supplementary material 5), our simulations reveal that future changes will depend on the level of warming, the decline being less intense under RCP2.6. We predicted a stability—or even an increase—in the EEZs ESI of the United Kingdom and Norway: a potential reallocation of fish stocks between fishery management zones is likely to occur in the coming decades, under all climate scenarios, under assumption of uniform spatial distribution of catches in EEZs.

## Discussion

### Temperature and primary production shaping species spatial distribution

Based on the concept of the ecological niche sensu Hutchinson (1978), our models rely on the ecological requirements of each species, mean Sea Bottom Temperature (SBT), SBT range -a proxy for temperature seasonality—and primary production being the variables that best reproduced the contemporary spatial distribution of Species in the Mediterranean and European Seas (Atlantic coasts of Spain, France and England). Temperature is a key factor for the life cycle of all aquatic animals, particularly for ectotherms, and especially for species that have a pelagic larval/recruitment phases^25^. Our results show that the spatial distribution of the studied species in the Mediterranean and European Seas (Atlantic coasts of Spain, France and England) is strongly explained by variability in seasonal SBT. Coastal Mediterranean fish abundance, including the eight species we considered here, can be influenced by temperature seasonality in coastal^26^ and deep zones^27^. The effects of temperature variations on species depend upon the timing of life cycle, the intensity and duration of exposure, as well as the speed at which changes in temperature occur. Acute short-term variations of temperature might have drastic, often detrimental, effects on fish physiology, whereas long-term gradual variations can lead to potential acclimation, through variations in metabolic and feeding behavior^28^. Adaptations are also likely to occur especially on the long-term, including evolutionary responses, acclimation or changes in movement or behavior^3^. At the individual level, genetic adaptation was an observed response^3^. Unless adaptation or acclimation can track the rate of warming, it is likely that stocks will be affected, both directly through individual physiological tolerances, and indirectly through climate-related changes to the abundance of prey, predators, competitors and pathogens^29^.

Primary production sustains the whole marine food chain and provides most of the endosomatic energy needed for heterotrophic species: previous studies have shown that 8% of the worldwide aquatic primary production (but ~ 25% for shelf ecosystems^30^) is required to sustain fisheries at the global scale^31^. Although obtained from estimations integrated within the upper water column (0–30 m depth), our simulations show that the inclusion of a proxy for food concentration is important to assess the distributional range of species. While the eight species we modelled are carnivores with trophic levels ranging from 3.2 (surmullet^32^) to 4.4 (European hake^33^), the indirect relationship between primary production and fish stocks has already been thoroughly documented^34^. The relationship between primary production and upper trophic levels is also strongly influenced by factors related to the trophic processes that define the movement of endosomatic energy along the food chain, but also by other physical factors such as chlorophyll a^35^, oxygen concentration, or other oceanographic variables^2^. Incorporating trophodynamic in species distribution models such as the direct/indirect biotic and/or trophic interactions (e.g., prey/predator relationships)—is needed to infer their relative contribution to community structure and dynamics, or to quantify the regulating effects of upper (or lower) trophic levels by bottom-up (or top-down) forces^36^. However, their integration in modelling frameworks is still a methodological challenge^8^.

### Northward species range shift distribution

Climate-induced changes in the distribution of fish communities have been described in several marine ecosystems^37^. Our simulations show a future range shift, from the Mediterranean Sea to the North European coasts, in the distribution of all species for all levels of warming. In the Mediterranean Sea, a high decline in the ESI of the species is expected by the end of the century under a pronounced warming (RCP8.5), while a potential temperature-induced limitation is expected to slow down the decline rate in ESI by at least 20% under scenarios RCP2.6 or RCP4.5. The predicted decline in ESI in the Mediterranean Sea is likely to be accompanied by an increase in ESI along the North European coasts. From a fishery management point of view, assuming that spatial distribution of catches is uniform in each EEZ, our results reveal that the Mediterranean countries, catches of all species could considerably decrease by the end of the century as climate warms (up to + 3.2 °C under scenario RCP 8.5) as revealed by the decrease in ESI of species; North European countries will benefit of stable or increasing species’ catch. This is in agreement with findings of^38^ where common sole, red mullet, European hake and European seabass distribution will be negatively affected by rising temperature. The magnitude of range shifts in species distributions in the Mediterranean Sea may deeply affect ecosystem functioning and economic activities related to fishing^39^. Similarly, the spatial range of all species is expected to shrink whatever the scenario and the future time period. Fisheries management adaptations to climate change should urgently consider these predictions, as the rapid decrease in covered area (period 2030–2039, e.g., for the anglerfishes, the gilthead seabream or the European seabass), may induce a rapid and non-reversible change in fisheries resources. Mid- and long-term projections highlight that the loss in the spatial extent of species is higher when the warming becomes severe (RCP8.5 versus RCP2.6/RCP4.5). This is in phase with^5^ which quantify the benefits to marine fisheries—and related economic outcomes^40^—of limiting global warming to 1.5 °C above preindustrial level.

### Fisheries and aquaculture management implications

Here, we highlight the importance of simulating long-term changes in fisheries under several climate change scenarios, especially in the context of uncertain future outcomes for food and nutritional security^41^. Rising temperature, pollution and overfishing are weakening the resilience of fisheries and fish stocks to climatic stressors^38^. Overfishing and climate change can be considered as the “biggest threats” that fisheries are facing^42^. Overfishing decreases fish stocks resilience to climate change by disturbing the food web structure and causing habitats destruction^42^. Considering the combined threats on fisheries, developing and implementing fisheries management climate-adaptive strategies that can help address shifts in species distribution (under spatial uniformity assumptions) can be of interest to help adapting to climate change, in particular through change in commercially-targeted species, spatial reallocation of fishing effort, improvement of fishing techniques and engines, or the implementation of fishing rights based on historical stock distribution^43^. Transformative adaptation of current fisheries and aquaculture, as well as their management, is urgently needed, especially for the most vulnerable countries such as northern African countries where socio-economic exposure, vulnerability and risk to climate change are high in comparison to European countries^6^. While aquaculture has been suggested as an alternative to the dramatic decline in Mediterranean and Black Sea fisheries^44^, our simulations detect that the two most farmed Mediterranean fish—*i.e.,* the European seabass and the gilthead seabream—may also be impacted by warming by the mid/end of the twenty-first century, with a reduction in the potential for coastal aquaculture suitable sites. Assessment of the impact of climate change on Mediterranean coastal aquaculture is yet to be developed, however, to consider a large panel of abiotic^45^ and biotic factors including the risk of increasing disease outbreaks^46^, as well as regional economic peculiarities such as heterogeneity in national economies, national food self-sufficiency and human habits for foods.

Because of the high sensitivity and exposure of Mediterranean fisheries to climate change^6^, coordinated actions and mitigation activities must be undertaken to stem the repercussions of the ongoing decline in marine resources. As a way of adaptation, changes to the food commodity market and/or its diversification, through the commercialization of lower economic value and/or non-indigenous fish species must be considered^47^: in the eastern and central Mediterranean Sea, respectively, marbled rabbitfish *Siganus rivulatus* and the blue crab *Callinectes sapidus* are now commercially exploited^48^.

To conclude, our study predicted the potential decline in demersal fish species distribution in the Mediterranean Sea and their potential reallocation in the North European coasts, under different RCP scenarios and three time period. Whatever the future warming conditions in the upcoming decades, an adaptation of the fisheries and aquaculture strategies is urgently needed, for all countries, and mostly, the most vulnerable ones. We therefore support further initiatives aiming to predict the ecological and economic consequences of climate change on the fisheries and aquaculture, at the Mediterranean and European Seas scale.

## Methods

### Input data

#### Occurrence data collection

For the eight species, we compiled contemporary occurrence data from three available public databases: The Ocean Biogeographic Information System (OBIS, http://www.iobis.org), the Global Biodiversity Information Facility (GBIF, https://www.gbif.org) and Fishbase (http://www.fishbase.org). Occurrence data may be collected from various sources (e.g., scientific surveys, on-board observers, geo-referenced fisheries catch or diving observations), independently of the sampling protocol (e.g. gear, mesh size). However, because such data are not mainly based on scientific surveys (e.g. MEDITS, ICES trawling surveys), they may suffer from spatial heterogenous sampling effort (e.g. due to accessibility or survey equipment^49^), potentially leading to a risk of spatial niche truncation (*i.e.,* when only a subset of the environmental conditions experienced by a species across its full range is characterized^18^). To alleviate this risk and build the most up-to-date datasets, (i) we also retrieved all available species observations from the literature (Supplementary Material 1), especially in limiting environmental conditions (i.e. at the edge of the environmental niche) and (ii) evaluated the species response curves to detect any niche truncation (see “Species distributions models and environmental variables” and “Ensemble model selection”). The resulting occurrence data ranged from 1950 to 2017. Recent records (*i.e.,* since 1990) represented 72.82 ± 6.12% of the total species occurrences. Past records (*i.e.,* before 1990) represented 10.79 ± 3.7% of the total species occurrences. Undated—and therefore discarded—species occurrences represented 16.37 ± 4.58%) of the total species occurrences. To avoid a biased estimation of the niche due to low quality occurrence records^50^, past or undated occurrence were only kept along the distribution edge when confirmed by recent records from the literature (Supplementary Material 6).

For each species, we preprocessed the data and improved the quality of the eight occurrence record datasets by removing (i) unreliable observations—such as preserved specimen- and incorrect taxonomic identifications, (ii) duplicate occurrences and (iii) locational errors, such as geographical outliers. The resulting number of observations ranged from 1211 for gilthead seabream to 15,827 for common sole. For each species, we aggregated occurrences on a 0.1° × 0.1° spatial grid (from 70°N to 70°S and from 180°E to 180°W) that corresponds to the resolution of the preprocessed environmental variables (see “ Environmental filter and pseudo-absence selection”).

#### Environmental variable pre-treatment

To model the contemporary (1990–2017) spatial distribution of species, we considered Sea Bottom Temperature (SBT), Sea Surface Temperature (SST), salinity (SSS), primary production (PP), bathymetry and distance-to-coast (Table 3). For all parameters, except bathymetry and distance-to-coast, we calculated a yearly averaged climatology for the period 1990–2017. Contemporary environmental variables were then bilinearly interpolated at a 0.1° × 0.1° spatial resolution in the geographical domain ranging from 70°N to 70°S and from 180°E to 180°W to match the spatial resolution and extent of occurrence data.

**Table 3:** Contemporary and future (from General Circulation Models; GCMs) environmental variables used in this study.

Environmental variable	Contemporary	Future
*Bathymetry: spatial seafloor depth (m)	Global seafloor topography (Smith and Sandwell 1997)	
*Distance to coast: distance to the nearest coast (km)	NASA Goddard Space Flight Center (2009) (https://oceancolor.gsfc.nasa.gov/docs/distfromcoast/)	
*SSS: sea surface salinity	Levitus’ climatology (Levitus 2011) completed with ICES data (http://www.ices.dk/)	
**SBT:** mean annual sea bottom temperature (°C)	CORA : Coriolis Ocean database for ReAnalysis (Cabanes et al. 2013)	IPSL-CM5A-LR (Dufresne et al. 2013, Hourdin et al. 2013), MPI-ESM-LR (Stevens et al. 2013, Giorgetta et al. 2013), CNRM-CM5 (Voldoire et al. 2013), HadGEM2-ES (Jones et al. 2011) and GISS-E2-R (Schmidt et al. 2014) models
**SBTr:** mean annual sea bottom temperature range (°C)		
**SBTvar:** mean monthly sea bottom temperature variance (°C)		
**SST:** mean annual sea surface temperature (°C)	AVHRR Very High Resolution Radiometer (Casey et al. 2010)	
**SSTr:** mean annual sea surface temperature range (°C)		
**SSTvar:** mean monthly sea surface temperature variance (°C)		
**PP:** Primary Production (mol C. m^−2^.s^−1^). Averaged from five general circulation models (IPSL, MPI, CNRM, HadGEM and GISS	IPSL-CM5A-LR (Dufresne et al. 2013, Hourdin et al. 2013), MPI-ESM-LR (Stevens et al. 2013, Giorgetta et al. 2013), CNRM-CM5 (Voldoire et al. 2013), HadGEM2-ES (Jones et al. 2011) and GISS-E2-R (Schmidt et al. 2014) models	

Sea Surface Temperature corresponds to the 30 m surface layer temperature. Sea Bottom Temperature corresponds to the 30 m bottom vertical layer down to a maximum depth of 500 m. *Environmental variable kept constant in time.

To prevent possible biases associated with multicollinearity and unnecessary model complexity^14^, the combination of environmental variables tested by the model considered a set of uncorrelated factors (*i.e.,* selecting only one variable among each set of intercorrelated factor; Pearson’s r > 0.7). To avoid model over-parametrization, bathymetry and distance-to-coast, were tested in a hierarchical filtering approach^19^. First, using information from Fishbase (http://www.fishbase.org), we applied a bathymetry filter, which corresponds to the observed depth range of each species from 150 m (*e.g.,* for the European seabass) to 1000 m (*e.g.,* for the anglerfish). The absence of coastal shelf in the Mediterranean may prevent identifying suitable environment for the species, so we also considered a 50 km distance-to-coast filter to the geographical cells outside the observed depth range of species to allow including near-coastal areas as suitable environment.

### Description of the modelling framework

#### Modelling algorithms

The contemporary (1990–2017) distributions of the eight species were estimated by means of the Environmental Suitability Index (ESI), a spatialized index ranging from 0 to 1 that reflects suitable environmental conditions, i.e., where a species can live and reproduce. We performed the ensemble modelling framework designed by^9,19,22^ in order to (i) reduce sampling biases (*e.g.* the use of the convex hull method to generate pseudo-absences), (ii) improve model evaluation, and (iii) quantify methodological uncertainties by incorporating a large range of techniques (using a multi-GCMs and multi-scenarios approach, we also considered uncertainties about future climate conditions^51^). By using an ensemble modelling procedure over a single algorithm, our framework quantifies the intra- and inter-algorithm uncertainty in the response of species to environmental variables and the potential consequences on both contemporary and future projections^52^. Our procedure identifies and retains the statistical algorithms that best reproduce observed spatial distributions among the following methods^53^: (i) the Non-Parametric Probabilistic Ecological Niche model (NPPEN), (ii) the Generalized Linear Model (GLM), (iii) the Generalized Additive Model (GAM), (iv) the Generalized Boosting Model (GBM), (v) the Artificial Neural Network (ANN), (vi) the Flexible Discriminant Analysis (FDA), (vii) the Multiple Adaptive Regression Splines (MARS) and (viii) the Random Forest (RF). Each algorithm was calibrated using the default parameters available in Biomod2, that correspond to the traditional parameters adapted for presence/pseudo-absence data (e.g. binomial distribution family (link = ‘logit’) for regression-based algorithms; see^53^ for details). For each algorithm and species, we performed a 10-time random cross-validation run, training each algorithm on 70% of the data and keeping the remaining 30% for evaluation-only.

#### Environmental filter and pseudo-absence selection

Spatially biased sampling effort in presence-only species datasets—*i.e.,* when data sources are not comprehensive across the study area—can induce a bias in the environmental space in which the spatial distribution of species is modelled^54^. To consider this potential effect for each species, we used an environmental filter to keep only a single observation among a group of occurrences characterized by a similar combination of environmental values (Supplementary Material 3), as performed in the GARP (Genetic Algorithm for Range Prediction) modelling system (program RASTERIZ^55^). We determined the following resolution for environmental filtering: 0.5 °C for temperature-related variables, 0.5 psu for SSS and 0.5 mol C.m^−2^.s^–1^ for PP (used in logarithm). The same environmental domain was used to generate pseudo-absences outside the space delimiting environmental suitable conditions using the convex hull method^56^ while excluding the 2.5 and 97.5 percentiles. The latter is defined as the smallest convex hyper-volume in the environmental space, containing occurrences points within the 2.5 and 97.5 percentiles for each environmental parameter (i.e. alleviating the weight of environmental extremes corresponding to first records). Pseudo-absences are then randomly generated in equal numbers to filtered presences, in the environmental space outside this convex hyper-volume, therefore minimizing prediction variance (see "D-designs” theory^57^)This procedure alleviates model over-prediction and biases associated with heterogenous or discontinuous sampling effort, increasing therefore the ability of the model to mirror the observed distributional range^58^.

#### Ensemble model selection

For each species and combination of environmental variables, the algorithms that best reflected the observed distribution were selected according to the Continuous Boyce Index (CBI), an evaluation metric specifically designed for presence/pseudo-absence datasets. We retained algorithms with a CBI value over 0.5^58^. To ensure the ecological realism of our models, we discarded spurious responses to environmental factors (*e.g.,* bimodal response to temperature) and selected the simulations for which response curves matched a priori expectations (see^22^ for further details). The inter-algorithm divergence in responses curves (i.e. a major criticism of ensemble modelling procedure^19^ have been quantified by means of the standard deviation (SD) computed from all retained simulations (Figure S2. in the supplementary material 2).

### Future projections

#### Time scales and climatic scenarios

Following a multi-GCMs and multi-scenarios approach to evaluate the potential future distributions of the eight species while considering uncertainties about future climate conditions, we retrieved information from five high-resolution General Circulation Models (IPSL-CM5A-LR, MPI-ESM-LR, CNRM-CM5, HadGEM2-ES, GISS-E2-R; see references in Table 1) and three RCP scenarios from the 5th Coupled Model Intercomparison Project (CMIP5) according to the radiative forcing: the low RCP2.6, the medium–low RCP4.5, and the high RCP8.5. To alleviate inter-annual stochasticity in species distributions and to highlight the main patterns of changes, we averaged future temperature-related variables and PP for three different decades: 2030–2039 (short-term projections), 2050–2059 (mid-term projections) and 2090–2099 (long-term projections). Future SSS was considered constant in time as the temporal variations are known to be negligible^59^ in contrast to spatial variations (*i.e.* discriminate marine from brackish waters). To match the spatial resolution and extent of contemporary environmental variables, we bilinearly interpolated future environmental variables at a 0.1° × 0.1° spatial resolution and in the geographical domain ranging from 70°N to 70°S and from 180°E to 180°W.

#### Pre-treatment of future temperature data

To assess possible bias between contemporary and future temperature-related variables, we performed Taylor diagrams^60^ to estimate the consistency between current and future climate data (Supplementary Material 7): considering a common period (*i.e.* 2006–2017), we calculated the Pearson correlation coefficient, the Root-Mean-Square Difference (RMSD) and the standard deviation (SD) difference for each temperature-related variable. For each GCM and RCP scenario, we then corrected model-based temperature data according to their difference with observation-based data for each geographical cell. This procedure ensured a perfect correlation (Pearson coefficient r = 1), no RMSD and the same SD between model- and observation-based datasets for a common period^61^.

### Projected changes in species environmental suitability index

For each future period, we estimated the occurrence of each species per geographical cell (0.1° × 0.1°) by combining our ensemble modelling method with environmental data originating from the five GCMs and the three RCP scenarios. For each species and future period, we calculated the proportion (in km^2^) of the studied area that was projected to contain a suitable habitat in order to quantify (as percentage) potential changes in the spatial extent of species, relative to the contemporary (1990–2017) period. As an index of the potential consequences of distributional shifts at the scale of each Mediterranean and European EEZs—we assessed the potential consequences of distributional shifts at the scale of each Mediterranean and European EEZs under the assumption of uniform spatial distribution of catches in EEZs. We calculated ESI by EEZ as the mean by pixel (0.1° × 0.1°) in each EEZ^62^, stretching from the coastline out to 200 nautical miles over which a country has special rights regarding the use of marine resources. We also calculated the total catch landings for each species over the period 1990–2017 (in logarithm). For each species and EEZ, we downloaded the mean catch data from the Sea Around Us database (http://www.seaaroundus.org/) for the period 1990–2017 (i.e., the most recent available information). Focusing on EEZs allowed the estimation of changes at the scale of basic units for fisheries management (*e.g.* attribution of maximum allowed catches by EEZs) and conservation perspectives^62^. In addition, EEZs are relevant regions for biogeographic research, and are commonly investigated for assessing the socio-economic consequences of climate change on fisheries^5^.

## Supplementary Information

Supplementary Information.

## Data Availability

These data were derived from the following resources available in the public domain: the Ocean Biogeographic Information System Mapper (OBIS, http://www.iobis.org/mapper/), the Global Biodiversity Information Facility (GBIF, https://www.gbif.org/) and Fishbase (http://www.fishbase.org/).
