# Supplementary material for: Expected contraction in the distribution ranges of demersal fish of high economic value in the Mediterranean and European Seas
Source: Sci Rep. 2022 Jun 16;12:10150. doi: 10.1038/s41598-022-14151-8 (PMC9203508; doi:10.1038/s41598-022-14151-8)
Supplement: Supplementary file 1 — Supplementary Information. [file 41598_2022_14151_MOESM1_ESM.pdf]

Supplementary material

Title : Expected contraction of the distribution ranges of demersal fish of high economic value in the Mediterranean and European Seas

Emna Ben Lamine[1,2]*, Alexandre Schickele[1,3], Eric Goberville[4], Gregory Beaugrand[5], Denis Allemand[2,6] & Virginie Raybaud[1,2]

*Corresponding author, https://orcid.org/0000-0002-9386-0186

[1] Université Côte d'Azur, CNRS, UMR 7035 ECOSEAS, Nice, France

[2] LIA ROPSE, Laboratoire International Associé Université Côte d'Azur - Centre Scientifique de Monaco, Monaco

[3] Sorbonne Université, CNRS, UMR 7093 LOV, Villefranche-sur-Mer, France

[4] Muséum National d'Histoire Naturelle, Sorbonne Université, Université de Caen Normandie, Université des Antilles, CNRS, UMR 8067 BOREA, Paris, France.

[5] Université Lille, Université Littoral Côte d'Opale, CNRS, UMR 8187 LOG, Wimereux, France.

[6] Centre Scientifique de Monaco, Monaco, Monaco

Supplementary material 1. **Table s1**. Evaluation metrics for all environmental parameter's combinations. White cells correspond to algorithms with a CBI<0.5. Cells with "x" correspond to algorithms with a CBI>0.5 with a relevant response curve. Grey cells indicate that only one model resulted in a relevant response curve.

		Seleted models								Mean Evaluation metric				
**Species**	**Evrironmental parameters**	**GLM**	**GBM**	**GAM**	**ANN**	**RF**	**FDA**	**MARS**	**NPPEN**	**CBI**	**AUC**	**TSS**	**Sorense**	**Jaccard**
**Anglerfish**	SST_mean. SST_range. Salinity	x			x				x	0.75	0.816	0.514	0.786	0.847
	SST_mean. SST_var. Salinity	x			x				x	0.722	0.854	0.877	0.797	0.862
	SST_mean. SST_range. log_PP				x				x	0.7945	0.71	0.746	0.774	0.872
	SST_mean. SST_var. log_PP				x				x	0.749	0.886	0.695	0.741	0.85
	SBT_mean. SBT_range. Salinity	x		x					x	0.759	0.952	0.533	0.861	0.852
	SBT_mean. SBT_var. Salinity			x	x				x	0.777	0.952	0.706	0.776	0.874
	SBT_mean. SBT_range. log_PP				x		x	x	x	0.816	0.958	0.716	0.796	0.668
	SBT_mean. SBT_var. log_PP	x			x				x	0.736	0.964	0.723	0.832	0.785
	SBT_max. SBT_range. salinity	x			x				x	0.802	0.923	0.726	0.801	0.885
	SBT_max. SBT_var. salinity				x				x	0.7735	0.935	0.848	0.87	0.878
	SBT_min. SBT_range. salinity				x		x		x	0.666	0.949	0.723	0.814	0.894
	SBT_min. SBT_var. salinity	x			x				x	0.76466	0.953	0.827	0.701	0.843
	SBT_max. SBT_range. log_PP								x					
	SBT_max. SBT_var. log_PP			x					x	0.8115	0.952	0.706	0.776	0.874
	SBT_min. SBT_range. log_PP						x	x	x	0.777	0.896	0.832	0.853	0.952
	SBT_min. SBT_var. Log_PP			x				x	x	0.68666	0.958	0.716	0.796	0.668
	SBT_mean. Salinity. log_PP				x				x	0.641	0.964	0.723	0.832	0.785
**European hake**	SST_mean. SST_range. Salinity	x			x			x	x	0.76525	0.98	0.85	0.93	0.87
	SST_mean. SST_var. Salinity	x			x			x	x	0.7235	0.98	0.86	0.93	0.88
	SST_min. SST_range. Salinity	x			x				x	0.68733	0.97	0.84	0.93	0.86
	SST_min. SST_var. Salinity	x			x			x	x	0.7615	0.98	0.84	0.92	0.86
	SST_max. SST_range. Salinity	x			x				x	0.72633	0.97	0.85	0.93	0.87
	SST_max. SST_var. Salinity	x			x				x	0.71833	0.98	0.88	0.94	0.89
	SST_mean. SST_range. log_PP					x		x	x	0.83566	0.964	0.723	0.832	0.785
	SST_mean. SST_var. log_PP					x			x	0.7675	0.98	0.85	0.93	0.87
	SST_min. SST_range. log_PP							x	x	0.791	0.98	0.86	0.93	0.88
	SST_min. SST_var. log_PP							x	x	0.764	0.97	0.84	0.93	0.86
	SST_max. SST_range. log_PP						x		x	0.725	0.98	0.84	0.92	0.86
	SST_max. SST_var. log_PP							x	x	0.6325	0.97	0.85	0.93	0.87
	SBT_mean. SBT_range. Salinity	x						x	x	0.74166	0.98	0.88	0.94	0.89
	SBT_mean. SBT_var. Salinity	x			x				x	0.81866	0.97	0.84	0.93	0.86
	SBT_mean. SBT_range. log_PP			x				x	x	0.87966	0.98	0.84	0.92	0.86
	SBT_mean. SBT_var. log_PP							x	x	0.7635	0.97	0.85	0.93	0.87
	SBT_max. SBT_range. salinity	x			x				x	0.657	0.935	0.848	0.87	0.878
	SBT_max. SBT_var. salinity	x			x				x	0.652	0.949	0.723	0.814	0.894
	SBT_min. SBT_range. salinity	x			x				x	0.65233	0.953	0.827	0.701	0.843
	SBT_min. SBT_var. salinity	x		x					x	0.737	0.959	0.695	0.771	0.864
	SBT_max. SBT_range. log_PP						x	x	x	0.69233	0.952	0.706	0.776	0.874
	SBT_max. SBT_var. log_PP			x				x	x	0.646	0.951	0.832	0.85	0.915
	SBT_min. SBT_range. log_PP			x			x		x	0.82866	0.958	0.716	0.796	0.668
	SBT_min. SBT_var. Log_PP						x		x	0.7605	0.911	0.585	0.676	0.706
	SBT_mean. Salinity. log_PP	x							x	0.661	0.854	0.359	0.748	0.508

Species	Evrironmental parameters	Seleted models								Mean Evaluation metric				
		GLM	GBM	GAM	ANN	RF	FDA	MARS	NPPEN	CBI	AUC	TSS	Sorense	Jaccard
Common Sole	SST_mean. SST_range. Salinity				x		x		x	0.76466	0.956	0.726	0.625	0.895
	SST_mean. SST_var. Salinity	x					x	x	x	0.77675	0.816	0.514	0.786	0.847
	SST_mean. SST_range. log_PP	x						x	x	0.745	0.854	0.877	0.797	0.862
	SST_mean. SST_var. log_PP	x			x		x		x	0.7375	0.71	0.746	0.774	0.872
	SBT_mean. SBT_range. Salinity			x	x				x	0.71933	0.886	0.695	0.741	0.85
	SBT_mean. SBT_var. Salinity				x				x	0.743	0.911	0.585	0.676	0.706
	SBT_mean. SBT_range. log_PP				x		x	x	x	0.83575	0.854	0.359	0.748	0.508
	SBT_mean. SBT_var. log_PP			x				x	x	0.82766	0.956	0.726	0.625	0.895
	SBT_max. SBT_range. salinity	x			x				x	0.726	0.816	0.514	0.786	0.847
	SBT_max. SBT_var. salinity			x			x		x	0.79233	0.854	0.877	0.797	0.862
	SBT_min. SBT_range. salinity				x				x	0.6795	0.71	0.746	0.774	0.872
	SBT_min. SBT_var. salinity						x		x	0.796	0.886	0.695	0.741	0.85
	SBT_max. SBT_range. log_PP	x							x	0.79233	0.854	0.877	0.797	0.862
	SBT_max. SBT_var. log_PP								x					
	SBT_min. SBT_range. log_PP						x		x	0.796	0.886	0.695	0.741	0.85
	SBT_min. SBT_var. Log_PP				x			x	x	0.77466	0.796	0.716	0.796	0.915
	SBT_mean. Salinity. log_PP	x							x	0.691	0.98	0.84	0.92	0.86
European seabass	SST_mean. SST_range. Salinity	x			x				x	0.70433	0.97	0.85	0.93	0.87
	SST_mean. SST_var. Salinity	x		x				x	x	0.72125	0.935	0.848	0.87	0.878
	SST_mean. SST_range. log_PP						x	x	x	0.77833	0.949	0.723	0.814	0.894
	SST_mean. SST_var. log_PP				x		x	x	x	0.75825	0.953	0.827	0.701	0.843
	SST_mean. Salinity. log_PP		x		x		x		x	0.754	0.959	0.695	0.771	0.864
	SBT_mean. SBT_range. Salinity	x			x				x	0.74833	0.952	0.706	0.776	0.874
	SBT_mean. SBT_var. Salinity	x			x				x	0.718	0.98	0.84	0.92	0.86
	SBT_mean. SBT_range. log_PP				x			x	x	0.82266	0.97	0.85	0.93	0.87
	SBT_mean. SBT_var. log_PP				x			x	x	0.67633	0.935	0.848	0.87	0.878
	SBT_max. SBT_range. salinity	x			x				x	0.79266	0.949	0.723	0.814	0.894
	SBT_max. SBT_var. salinity	x		x	x				x	0.60775	0.953	0.827	0.701	0.843
	SBT_min. SBT_range. salinity	x			x				x	0.80333	0.959	0.695	0.771	0.864
	SBT_min. SBT_var. salinity	x			x				x	0.69366	0.952	0.706	0.776	0.874
	SBT_max. SBT_range. log_PP			x			x		x	0.816	0.854	0.832	0.85	0.915
	SBT_max. SBT_var. log_PP			x	x				x	0.75333	0.958	0.716	0.796	0.668
	SBT_min. SBT_range. log_PP				x		x		x	0.78333	0.949	0.723	0.814	0.894
	SBT_min. SBT_var. Log_PP			x				x	x	0.73533	0.953	0.827	0.701	0.843
	SBT_mean. Salinity. log_PP	x							x	0.7115	0.959	0.695	0.771	0.864
Gilthead seabream	SST_mean. SST_range. Salinity	x			x				x	0.80766	0.952	0.706	0.776	0.874
	SST_mean. SST_var. Salinity	x			x				x	0.80266	0.949	0.723	0.814	0.894
	SST_mean, SST_range, log_PP						x		x	0.7285	0.953	0.827	0.701	0.843
	SST_mean, SST_var, log_PP							x	x	0.6725	0.959	0.695	0.771	0.864
	SST_mean, Salinity, log_PP	x					x	x	x	0.6595	0.952	0.706	0.776	0.874
	SBT_mean, SBT_range, Salinity	x			x				x	0.802	0.956	0.726	0.625	0.895
	SBT_mean, SBT_var, Salinity	x			x				x	0.77766	0.816	0.514	0.786	0.847
	SBT_mean, SBT_range, log_PP				x		x	x	x	0.82375	0.854	0.877	0.797	0.862
	SBT_mean, SBT_var, log_PP						x		x	0.7195	0.71	0.746	0.774	0.872
	SBT_max, SBT_range, salinity			x					x	0.7925	0.886	0.695	0.741	0.85
	SBT_max, SBT_var, salinity	x			x				x	0.785	0.953	0.827	0.701	0.843
	SBT_min, SBT_range, salinity	x			x				x	0.77766	0.959	0.695	0.771	0.864
	SBT_min, SBT_var, salinity	x			x				x	0.75366	0.952	0.706	0.776	0.874
	SBT_max, SBT_range, log_PP			x			x	x	x	0.785	0.953	0.827	0.701	0.843
	SBT_max, SBT_var, log_PP				x		x		x	0.77766	0.959	0.695	0.771	0.864
	SBT_min, SBT_range, log_PP				x		x		x	0.80866	0.951	0.832	0.865	0.941
	SBT_min. SBT_var. Log_PP						x		x	0.7885	0.964	0.723	0.832	0.785
	SBT_mean. Salinity. log_PP	x					x		x	0.675	0.923	0.726	0.801	0.885

Species	Evrironmental parameters	Seleted models								Mean Evaluation metric				
		GLM	GBM	GAM	ANN	RF	FDA	MARS	NPPEN	CBI	AUC	TSS	Sorense	Jaccard
Surmullet	SST_mean. SST_range. Salinity	x						x	x	0.86766	0.952	0.706	0.776	0.874
	SST_mean. SST_var. Salinity	x		x					x	0.847	0.949	0.723	0.814	0.894
	SST_min. SST_range. Salinity	x			x				x	0.834	0.953	0.827	0.701	0.843
	SST_min. SST_var. Salinity	x						x	x	0.86533	0.959	0.695	0.771	0.864
	SST_max. SST_range. Salinity	x		x	x				x	0.8215	0.953	0.857	0.759	0.877
	SST_max. SST_var. Salinity	x					x		x	0.82166	0.911	0.828	0.845	0.915
	SST_mean. SST_range. log_PP				x				x	0.897	0.952	0.706	0.776	0.874
	SST_mean. SST_var. log_PP				x				x	0.716	0.911	0.585	0.676	0.706
	SST_min. SST_range. log_PP			x				x	x	0.773	0.854	0.359	0.748	0.508
	SST_min. SST_var. log_PP		x	x					x	0.81066	0.956	0.726	0.625	0.895
	SST_max. SST_range. log_PP							x	x	0.855	0.816	0.514	0.786	0.847
	SST_max. SST_var. log_PP							x	x	0.8595	0.854	0.877	0.797	0.862
	SBT_mean. SBT_range. Salinity	x			x				x	0.83366	0.71	0.746	0.774	0.872
	SBT_mean. SBT_var. Salinity	x			x			x	x	0.839	0.886	0.695	0.741	0.85
	SBT_mean. SBT_range. log_PP			x	x			x	x	0.868	0.816	0.514	0.786	0.847
	SBT_mean. SBT_var. log_PP			x	x			x	x	0.84025	0.854	0.877	0.797	0.862
	SBT_max. SBT_range. salinity	x			x				x	0.81033	0.71	0.746	0.774	0.872
	SBT_max. SBT_var. salinity	x		x					x	0.82366	0.886	0.695	0.741	0.85
	SBT_min. SBT_range. salinity	x					x		x	0.79733	0.911	0.828	0.845	0.915
	SBT_min. SBT_var. salinity	x			x				x	0.74733	0.952	0.706	0.776	0.874
	SBT_max. SBT_range. log_PP				x				x	0.862	0.958	0.716	0.796	0.668
	SBT_max. SBT_var. log_PP						x	x	x	0.779	0.964	0.723	0.832	0.785
	SBT_min. SBT_range. log_PP			x	x				x	0.83533	0.923	0.726	0.801	0.885
	SBT_min. SBT_var. Log_PP				x		x		x	0.82433	0.935	0.848	0.87	0.878
	SBT_mean. Salinity. log_PP	x							x	0.6845	0.958	0.716	0.796	0.668
Red mullet	SST_mean. SST_range. Salinity	x							x	0.917	0.911	0.585	0.676	0.706
	SST_mean. SST_var. Salinity	x		x			x		x	0.831	0.854	0.359	0.748	0.508
	SST_min. SST_range. Salinity	x			x		x		x	0.8365	0.956	0.726	0.625	0.895
	SST_min. SST_var. Salinity	x							x	0.813	0.816	0.514	0.786	0.847
	SST_max. SST_range. Salinity	x			x				x	0.87566	0.854	0.877	0.797	0.862
	SST_max. SST_var. Salinity	x							x	0.9035	0.71	0.746	0.774	0.872
	SST_mean. SST_range. log_PP								x					
	SST_mean. SST_var. log_PP							x	x	0.859	0.816	0.514	0.786	0.847
	SST_min. SST_range. log_PP							x	x	0.8345	0.854	0.877	0.797	0.862
	SST_min. SST_var. log_PP							x	x	0.848	0.71	0.746	0.774	0.872
	SST_max. SST_range. log_PP						x		x	0.713	0.886	0.695	0.741	0.85
	SST_max. SST_var. log_PP						x		x	0.7995	0.911	0.828	0.845	0.915
	SBT_mean. SBT_range. Salinity	x			x				x	0.812	0.952	0.706	0.776	0.874
	SBT_mean. SBT_var. Salinity	x			x				x	0.81966	0.958	0.716	0.796	0.668
	SBT_mean. SBT_range. log_PP			x			x	x	x	0.8565	0.964	0.723	0.832	0.785
	SBT_mean. SBT_var. log_PP						x		x	0.7995	0.923	0.726	0.801	0.885
	SBT_max. SBT_range. salinity	x			x				x	0.78566	0.935	0.848	0.87	0.878
	SBT_max. SBT_var. salinity	x			x		x		x	0.77825	0.816	0.514	0.786	0.847
	SBT_min. SBT_range. salinity	x		x	x				x	0.7915	0.854	0.877	0.797	0.862
	SBT_min. SBT_var. salinity	x							x	0.77825	0.71	0.746	0.774	0.872
	SBT_max. SBT_range. log_PP			x			x		x	0.78266	0.886	0.695	0.741	0.85
	SBT_max. SBT_var. log_PP				x			x	x	0.82033	0.911	0.828	0.845	0.915
	SBT_min. SBT_range. log_PP				x		x		x	0.72766	0.952	0.706	0.776	0.874
	SBT_min. SBT_var. Log_PP						x		x	0.718	0.958	0.716	0.796	0.668
	SBT_mean. Salinity. log_PP	x							x	0.7225	0.964	0.723	0.832	0.785
Common pandora	SST_mean. SST_range. Salinity				x				x	0.7075	0.923	0.726	0.801	0.885
	SST_mean. SST_var. Salinity				x		x		x	0.74666	0.935	0.848	0.87	0.878
	SST_mean. SST_range. log_PP						x	x	x	0.68833	0.949	0.723	0.814	0.894
	SST_mean. SST_var. log_PP				x		x		x	0.61033	0.953	0.827	0.701	0.843
	SBT_mean. SBT_range. Salinity	x						x	x	0.762	0.959	0.695	0.771	0.864
	SBT_mean. SBT_var. Salinity	x			x				x	0.74	0.953	0.857	0.759	0.877
	SBT_mean. SBT_range. log_PP			x			x	x	x	0.82775	0.911	0.828	0.845	0.915
	SBT_mean. SBT_var. log_PP			x			x	x	x	0.78825	0.952	0.706	0.776	0.874
	SBT_max. SBT_range. salinity				x				x	0.74	0.954	0.851	0.862	0.9254
	SBT_max. SBT_var. salinity	x			x		x		x	0.72025	0.958	0.716	0.796	0.668
	SBT_min. SBT_range. salinity			x	x				x	0.763	0.964	0.723	0.832	0.785
	SBT_min. SBT_var. salinity			x			x		x	0.70666	0.923	0.726	0.801	0.885
	SBT_max. SBT_range. log_PP								x					
	SBT_max. SBT_var. log_PP				x		x		x	0.735	0.71	0.746	0.774	0.872
	SBT_min. SBT_range. log_PP						x		x	0.7715	0.933	0.861	0.692	0.811
	SBT_min. SBT_var. Log_PP				x				x	0.761	0.939	0.842	0.77	0.921
	SBT_mean. Salinity. log_PP			x	x				x	0.73466	0.858	0.921	0.827	0.865

Supplementary material 2.

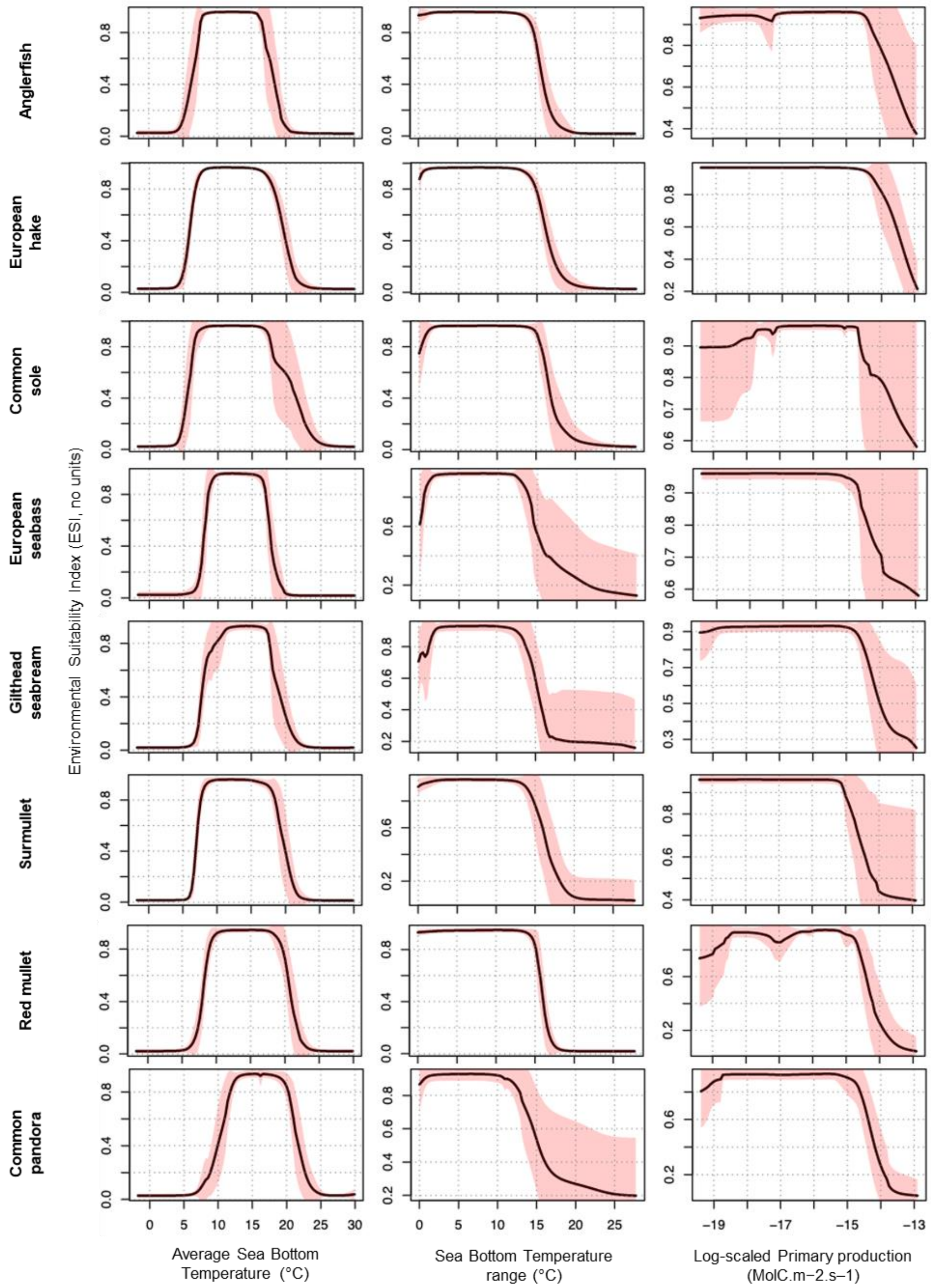

Figure s1. Species response to environmental variables. For each species, the black line represents the average ESI response to environmental variables, including all considered algorithms and cross-validation runs. The red area represents the corresponding standard deviation.

Supplementary material 3. Environmental data tests

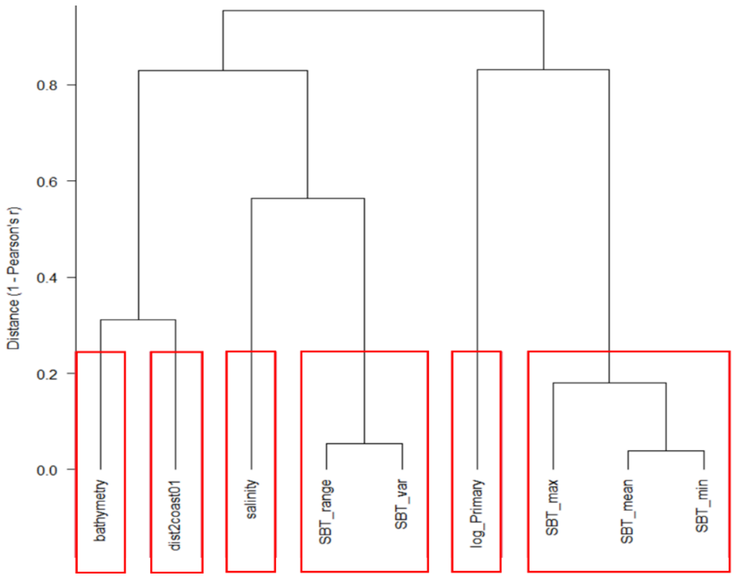

Figure s2. environmental parameter pre-selection process with groups of intercorrelated variables

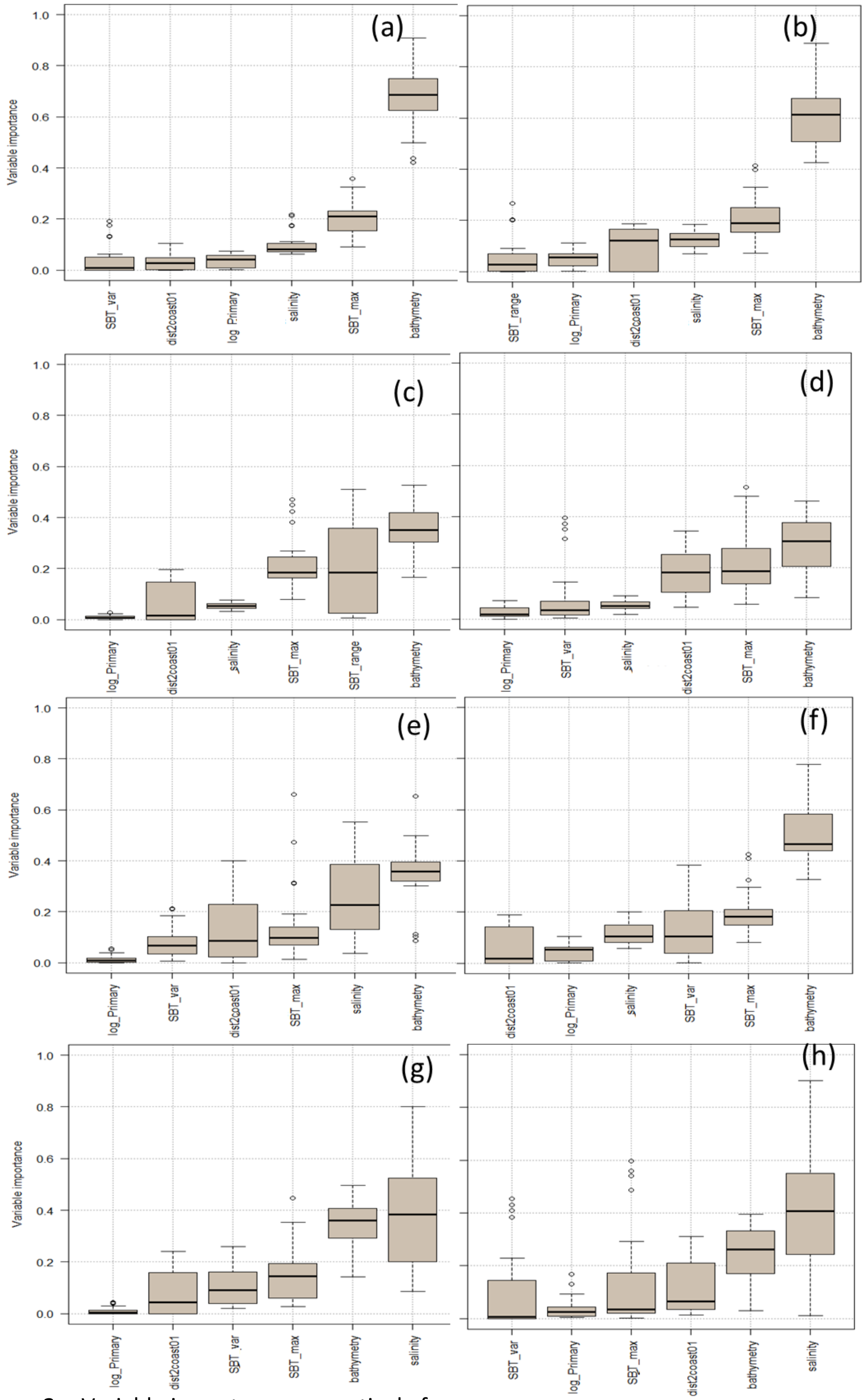

Figure s3 . Variable importance respectively for (a) Anglerfish, (b) European hake, (c) Common sole, (d) uropean seabass, (e) Gilthead seabream, (f) Surmullet, (g) Red mullet and (h) Common pandora. The methodological details concerning the variable pre-selection process presented here are adapted from Leroy et al. (2014) and Bellard et al. (2016) as both studies were used as a baseline for our modelling framework.

Supplementary material 4.

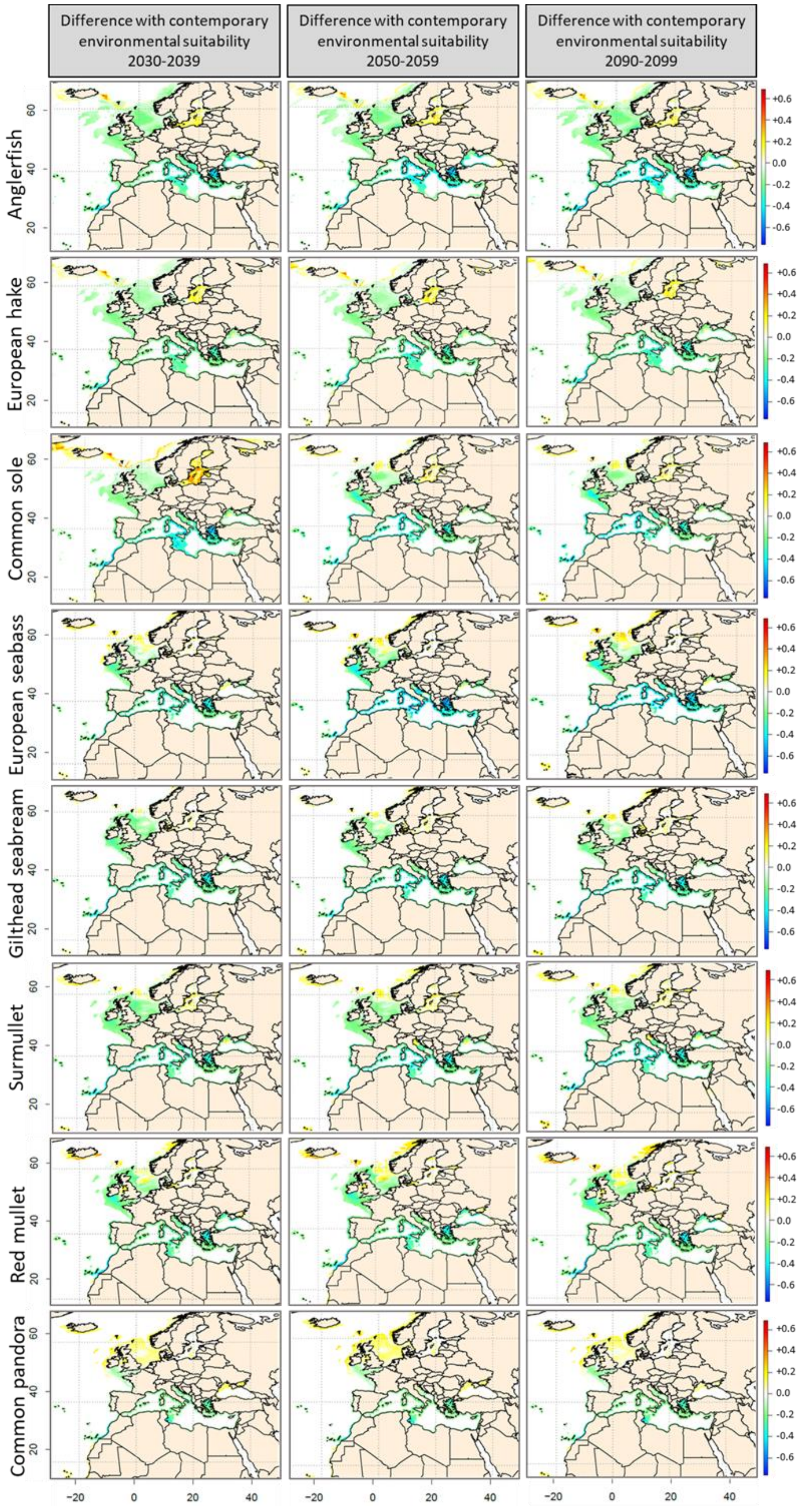

Figure s4. Differences in Environmental Suitability Index (ESI) values calculated between the current period (1990-2017) and the decade 2030-2039, 2050-2059 and 2090-2099 under scenario RCP2.6.

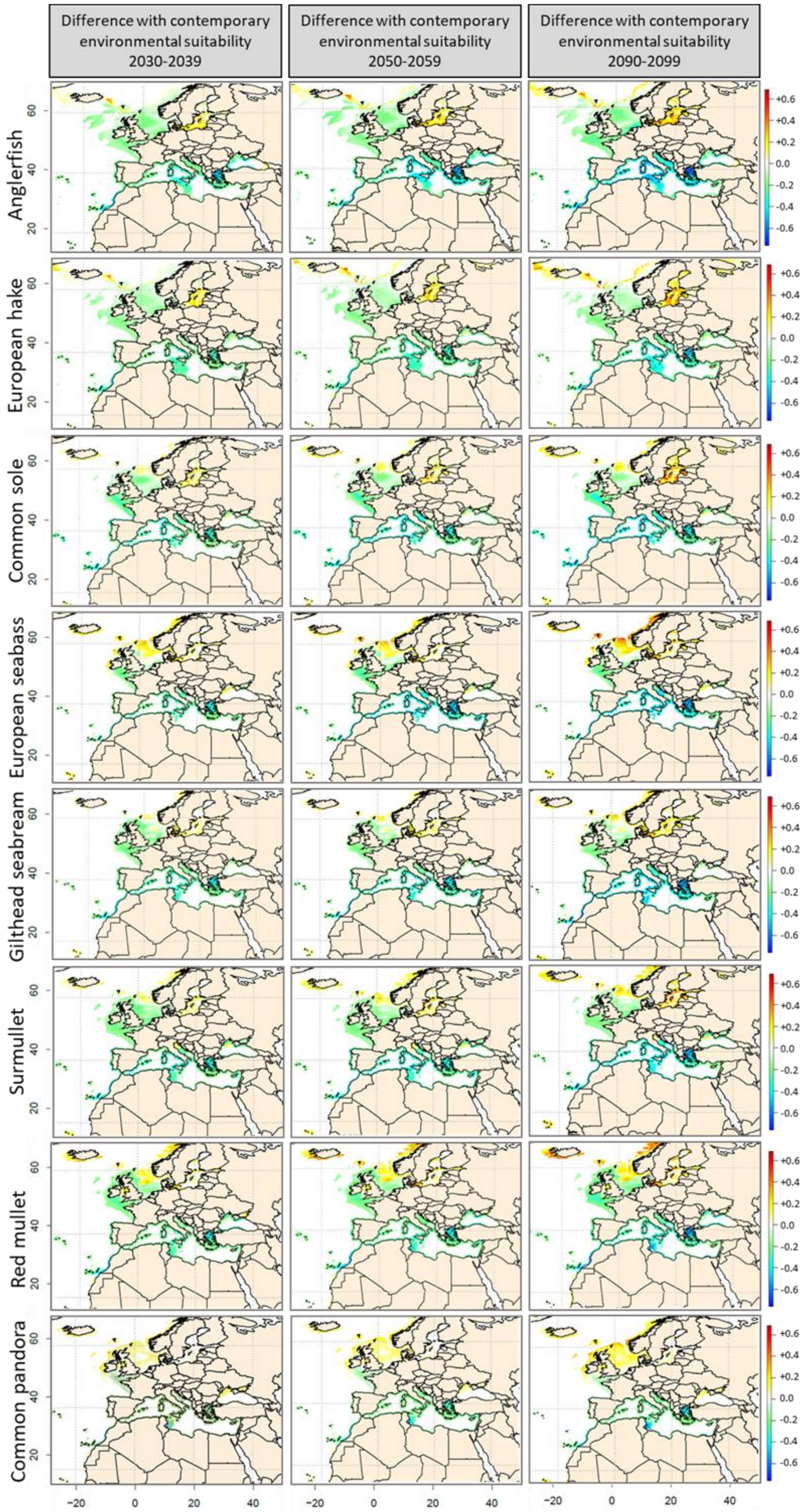

Figure s5. Differences in Environmental Suitability Index (ESI) values calculated between the current period (1990-2017) and the decade 2030-2039, 2050-2059 and 2090-2099 under scenario RCP4.5.

Supplementary material 5.

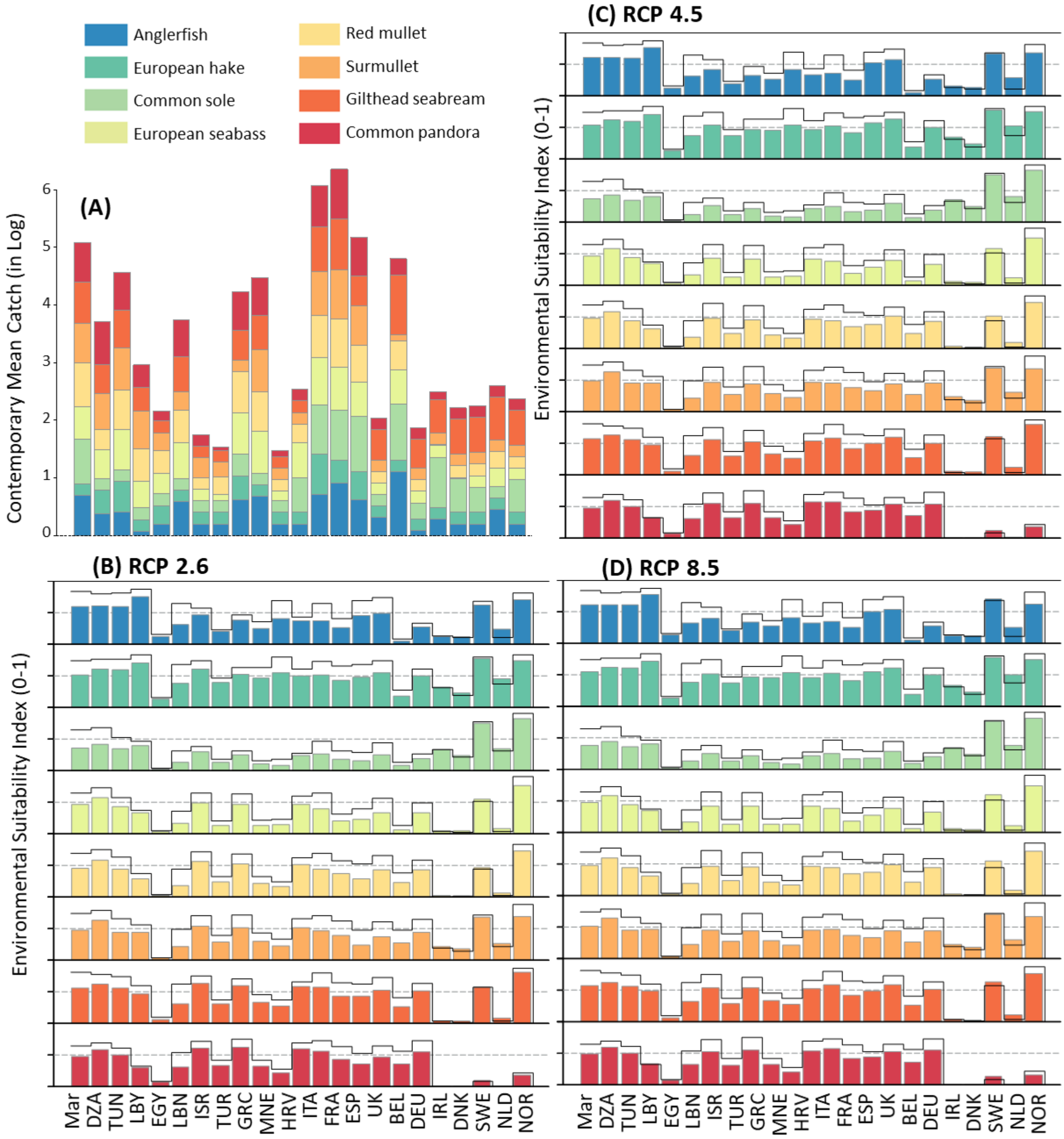

Figure s6. (A) Contemporary (1990- 2017) mean catch (in log) for the studied speciesin the Mediterranean Sea. (B-C-D) Projected changes in the Environmental Suitability Index (ESI) per Exclusive Economic Zone (EEZ) for each species, for the end of the century (2030-2039) under RCP 2.6 (B; bottom left), RCP 4.5 (C; top right) and RCP 8.5 (D; bottom right) scenarios. Bar plots for ESI are scaled from 0 to 1, the full black line corresponds to the ESI values for the current period (1990–2017) and colored bar correspond to the ESI values projected for 2090-2099. Countries with catches under 1000 tons per year are not shown. Countries are: MAR: Morocco, DZA: Algeria, TUN: Tunisia, LBY: Libya, EGY: Egypt, LBN: Lebanon, ISR: Israel; TUR: Turkey, GRC: Greece, MNE: Montenegro, HRV: Croatia; ITA: Italy, FRA: France, ESP: Spain, UK: United Kingdom, BEL: Belgium, DEU: Germany, IRL: Ireland, DNK: Denmark, SWE: Sweden, NLD: Netherlands, NOR: Norway

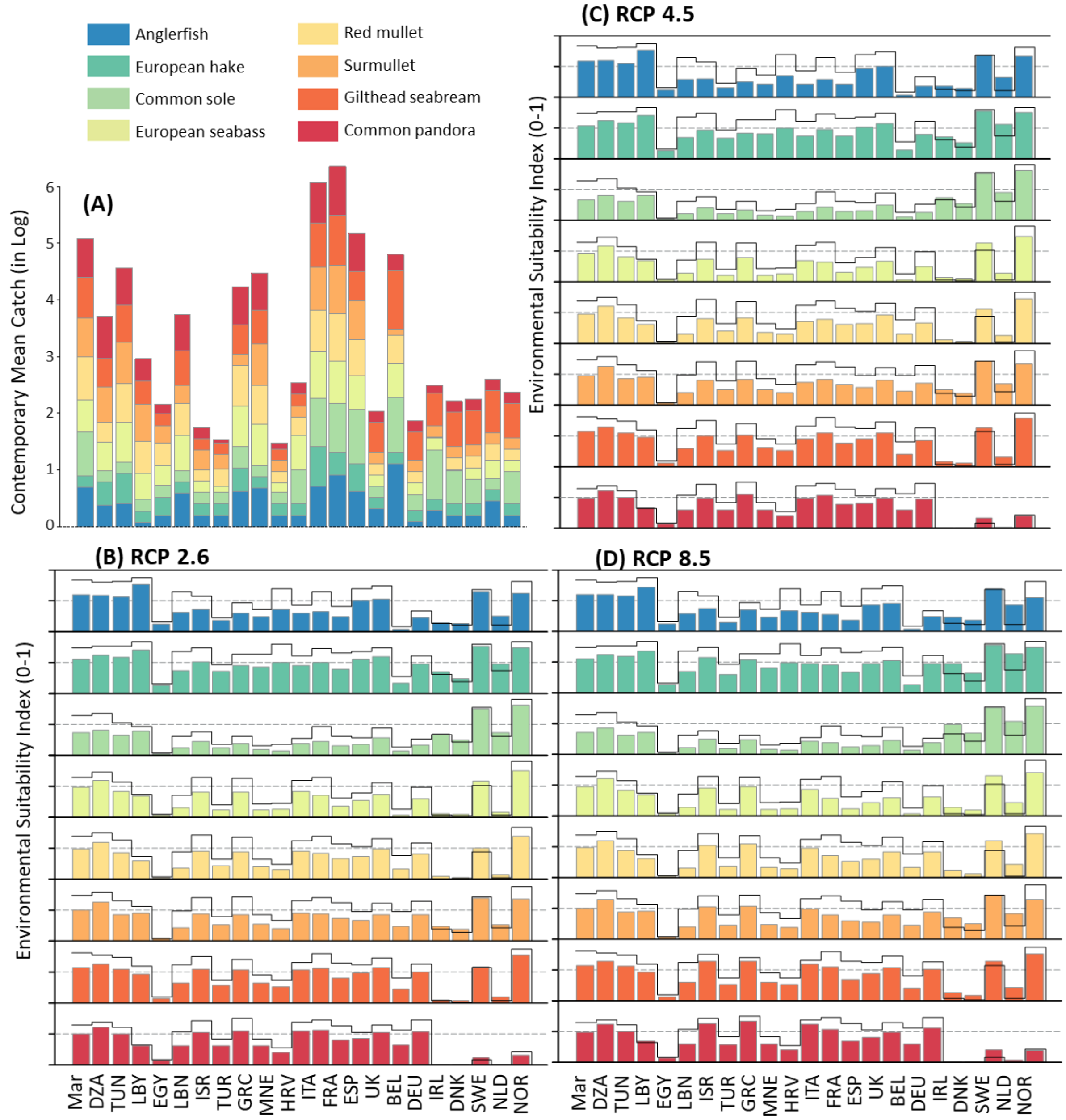

Figure s7. (A) Contemporary (1990- 2017) mean catch (in log) for the studied speciesin the Mediterranean Sea. (B-C-D) Projected changes in the Environmental Suitability Index (ESI) per Exclusive Economic Zone (EEZ) for each species, for the end of the century (2050-2059) under RCP 2.6 (B; bottom left), RCP 4.5 (C; top right) and RCP 8.5 (D; bottom right) scenarios. Bar plots for ESI are scaled from 0 to 1, the full black line corresponds to the ESI values for the current period (1990–2017) and colored bar correspond to the ESI values projected for 2090-2099. Countries with catches under 1000 tons per year are not shown. Countries are: MAR: Morocco, DZA: Algeria, TUN: Tunisia, LBY: Libya, EGY: Egypt, LBN: Lebanon, ISR: Israel; TUR: Turkey, GRC: Greece, MNE: Montenegro, HRV: Croatia; ITA: Italy, FRA: France, ESP: Spain, UK: United Kingdom, BEL: Belgium, DEU: Germany, IRL: Ireland, DNK: Denmark, SWE: Sweden, NLD: Netherlands, NOR: Norway

Supplementary material 6. **Table s2**. Detailed references used to complete the observations datasets

Species	References relative to observation data
**Anglerfishes**	Duarte et al., 2001; Barcala et al., 2020
**European hake**	Giani et al., 2019; Uzer et al., 2019
**Common sole**	Lepape et al., 2007; Vinagre et al., 2009
**European seabass**	Souche et al., 2015; Beraud et al., 2018
**Gilthead seabream**	Mercier et al., 2012; Žužul et al., 2019
**Surmullet**	Fabi et al., 2002; Pulleiro-Potel et al., 2015
**Red mullet**	Esposito et al., 2014 ; Giani et al., 2019
**Common pandora**	Diaz et al., 1994; Özbilgin et al., 2012

References

Barcala, E., Bellido, J.M., Bellodi, A., Carbonara, P., Carlucci, R., Casciaro, L., Esteban, A., Jadaud, A., Massaro, A., Peristaki, P., Meléndez, M.J., Pérez Gil, J.L., Salmerón, F., Pennino, M.G., 2020. Spatio-temporal variability in the distribution pattern of anglerfish species in the Mediterranean Sea. Sci. Mar. 83, 129. https://doi.org/10.3989/scimar.04966.11A

Beraud, C., van der Molen, J., Armstrong, M., Hunter, E., Fonseca, L., Hyder, K., 2018. The influence of oceanographic conditions and larval behaviour on settlement success—the European sea bass Dicentrarchus labrax (L.). ICES Journal of Marine Science 75, 455–470. https://doi.org/10.1093/icesjms/fsx195

Diaz, C., Padrón, A.G., Frías, I., Hardisson, A., Lozano, G., 1994. Concentrations of Mercury in Fresh and Salted Marine Fish From the Canary Islands. Journal of Food Protection 57, 246–248. https://doi.org/10.4315/0362-028X-57.3.246

Duarte, R., Azevedo, M., Landa, J., Pereda, P., 2001. Reproduction of angler®sh (Lophius budegassa Spinola and Lophius piscatorius Linnaeus) from the Atlantic Iberian coast. Fisheries Research 13.

Esposito, V., Andaloro, F., Bianca, D., Natalotto, A., Romeo, T., Scotti, G., Castriota, L., 2014. Diet and prey selectivity of the red mullet, *Mullus barbatus* (Pisces: Mullidae), from the southern Tyrrhenian Sea: the role of the surf zone as a feeding ground. Marine Biology Research 10, 167–178. https://doi.org/10.1080/17451000.2013.797585

Fabi, G., Sbrana, M., Biagi, F., Grati, F., Leonori, I., Sartor, P., 2002. Trammel net and gill net selectivity for Lithognathus mormyrus (L., 1758), Diplodus annularis (L., 1758) and Mullus barbatus (L., 1758) in the Adriatic and Ligurian seas. Fisheries Research 54, 375–388. https://doi.org/10.1016/S0165-7836(01)00270-3

Giani, D., Baini, M., Galli, M., Casini, S., Fossi, M.C., 2019. Microplastics occurrence in edible fish species (Mullus barbatus and Merluccius merluccius) collected in three different geographical sub-areas of the Mediterranean Sea. Marine Pollution Bulletin 140, 129–137. https://doi.org/10.1016/j.marpolbul.2019.01.005

Le Pape, O., Baulier, L., Cloarec, A., Martin, J., Le Loc'h, F., Désaunay, Y., 2007. Habitat suitability for juvenile common sole (Solea solea, L.) in the Bay of Biscay (France): A quantitative description using indicators based on epibenthic fauna. Journal of Sea Research 57, 126–136. https://doi.org/10.1016/j.seares.2006.08.011

Mercier, L., Mouillot, D., Bruguier, O., Vigliola, L., Darnaude, A., 2012. Multi-element otolith fingerprints unravel sea−lagoon lifetime migrations of gilthead sea bream Sparus aurata. Mar. Ecol. Prog. Ser. 444, 175–194. https://doi.org/10.3354/meps09444

Özbilgin, H., Metin, G., Tosunoğlu, Z., Tokaç, A., Kaykaç, H., Aydın, C., 2012. Seasonal variation in the trawl codend selectivity of common pandora (Pagellus erythrinus): Seasonal variation in the selectivity of trawl codend. Journal of Applied Ichthyology 28, 194–199. https://doi.org/10.1111/j.1439-0426.2011.01920.x

Pulleiro-Potel, L., Barcala, E., Mayo-Hernández, E., Muñoz, P., 2015. Survey of anisakids in commercial teleosts from the western Mediterranean Sea: Infection rates and possible effects of environmental and ecological factors. Food Control 55, 12–17. https://doi.org/10.1016/j.foodcont.2015.02.020

Souche, E.L., Hellemans, B., Babbucci, M., MacAoidh, E., Guinand, B., Bargelloni, L., Chistiakov, D.A., Patarnello, T., Bonhomme, F., Martinsohn, J.T., Volckaert, F.A.M., 2015. Range-wide population structure of European sea bass *Dicentrarchus labrax*. Biol. J. Linn. Soc. 116, 86–105. https://doi.org/10.1111/bij.12572

Uzer, uğur, Öztürk, B., Yildiz, T., 2019. Age composition, growth, and mortality of European hake Merluccius merluccius (Actinopterygii: Gadiformes: Merlucciidae) from the northern Aegean Sea, Turkey. Acta Ichthyol. Piscat. 49, 109–117. https://doi.org/10.3750/AIEP/02465

Vinagre, C., Maia, A., Reis-Santos, P., Costa, M.J., Cabral, H.N., 2009. Small-scale distribution of Solea solea and Solea senegalensis juveniles in the Tagus estuary (Portugal). Estuarine, Coastal and Shelf Science 81, 296–300. https://doi.org/10.1016/j.ecss.2008.11.008

Žužul, I., Šegvić-Bubić, T., Talijančić, I., Džoić, T., Lepen Pleić, I., Beg Paklar, G., Ivatek-Šahdan, S., Katavić, I., Grubišić, L., 2019. Spatial connectivity pattern of expanding gilthead seabream populations and its interactions with aquaculture sites: a combined population genetic and physical modelling approach. Sci Rep 9, 14718. https://doi.org/10.1038/s41598-019-51256-z

Supplementary material 7. Consistency between current and future climate data

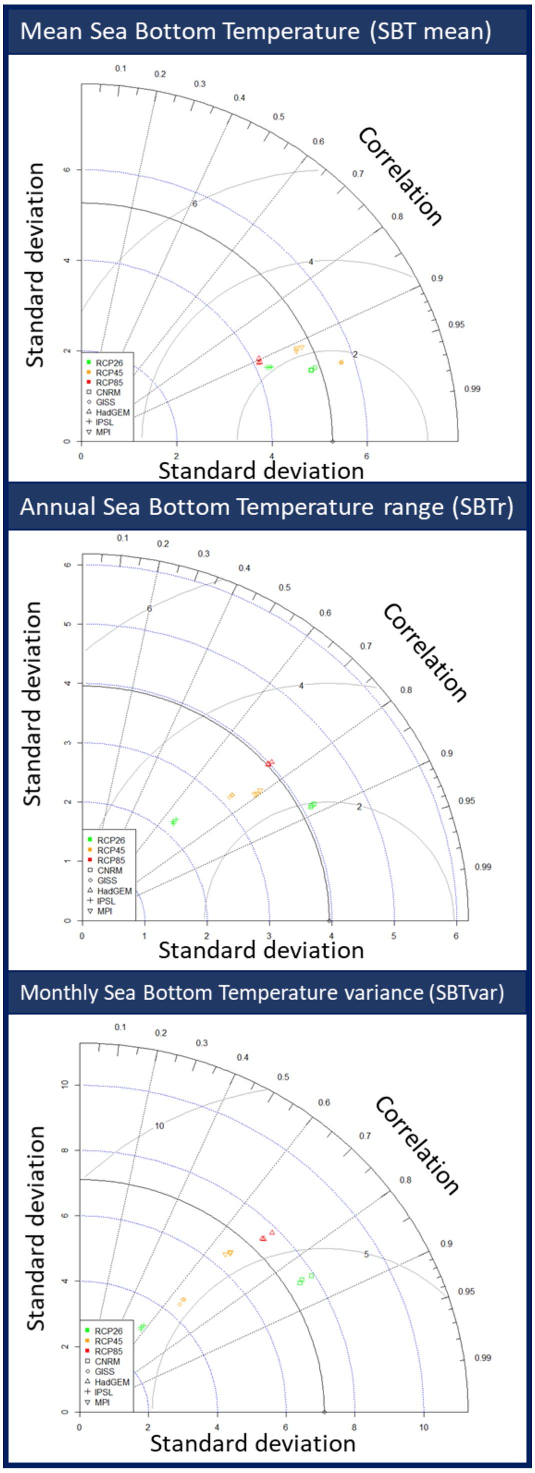

Figure S7. Taylor diagrams quantifying the difference between observation-based contemporary data (grey circle on the bottom of each diagram) and GCM-based (General Circulation Model) future climate conditions. for mean Sea Bottom Temperature (SBT), annual range of SBT, and monthly variance of SBT.
